# The oxygen-tolerant reductive glycine pathway assimilates methanol, formate and CO_2_ in the yeast *Komagataella phaffii*

**DOI:** 10.1038/s41467-023-43610-7

**Published:** 2023-11-27

**Authors:** Bernd M. Mitic, Christina Troyer, Lisa Lutz, Michael Baumschabl, Stephan Hann, Diethard Mattanovich

**Affiliations:** 1 University of Natural Resources and Life Sciences, Vienna, Department of Biotechnology, Institute of Microbiology and Microbial Biotechnology, Muthgasse 18, 1190 Vienna, Austria; 2 University of Natural Resources and Life Sciences, Vienna, Department of Chemistry, Institute of Analytical Chemistry, Muthgasse 18, 1190 Vienna, Austria; 3 Austrian Centre of Industrial Biotechnology (ACIB), Muthgasse 11, 1190 Vienna, Austria

**Keywords:** Metabolomics, Metabolic engineering, Applied microbiology, Metabolic engineering

## Abstract

The current climatic change is predominantly driven by excessive anthropogenic CO_2_ emissions. As industrial bioprocesses primarily depend on food-competing organic feedstocks or fossil raw materials, CO_2_ co-assimilation or the use of CO_2_-derived methanol or formate as carbon sources are considered pathbreaking contributions to solving this global problem. The number of industrially-relevant microorganisms that can use these two carbon sources is limited, and even fewer can concurrently co-assimilate CO_2_. Here, we search for alternative native methanol and formate assimilation pathways that co-assimilate CO_2_ in the industrially-relevant methylotrophic yeast *Komagataella phaffii* (*Pichia pastoris*). Using ^13^C-tracer-based metabolomic techniques and metabolic engineering approaches, we discover and confirm a growth supporting pathway based on native enzymes that can perform all three assimilations: namely, the oxygen-tolerant reductive glycine pathway. This finding paves the way towards metabolic engineering of formate and CO_2_ utilisation to produce proteins, biomass, or chemicals in yeast.

## Abstract (web summary)

One carbon compounds such as CO_2_, methanol and formate are cost-effective and environmentally friendly microbial feedstocks for biomanufacturing. Here, the authors report the oxygen tolerant reductive glycine pathway in *Komagataella phaffii* can co-assimilate CO_2_, methanol and formate.

## Introduction

The combustion of fossil fuels and the associated increase in atmospheric CO_2_ is the primary reason for anthropogenic climate change^1^. Regarding this global problem, CO_2_-fixation is of utmost importance. The use of CO_2_-derived renewable and green feedstocks as carbon sources for bioprocesses has increased in importance over the last years. This is not only because of climate change concerns, but also as a consequence of the problems emerging from food-competing bio-industrial feedstock, e.g., glucose. Green, non-food-competing carbon sources such as methanol (MeOH) and formate (FA) can be electrochemically produced from CO_2_^2,3^. Still, the number of organisms that can produce biomass, proteins, or chemicals from these green carbon sources is limited^4^.

*Komagataella phaffii* (also known as *Pichia pastoris*) is a methylotrophic yeast^5,6^ that is used industrially in the heterologous protein production of enzymes and biopharmaceuticals and the methanol-inducible alcohol oxidase promotor system^7–10^ of this yeast is well-known. Synthetic biology tools (e.g., CRISPR/Cas9) have simplified metabolic engineering of this methylotrophic host^11–14^, which was demonstrated by altering a heterotrophic organism in an auxotroph that can grow on CO_2_^15^. The main methanol assimilation pathway, the xylulose 5-phosphate pathway (XuMP), is well understood and has been investigated in detail^16^. A hitherto unknown metabolic route was recently discovered in *K. phaffii*. Although the flux is too low to support growth^17^, this route is active. In the synthetic autotrophic strain expressing a heterologous Calvin-Benson-Bassham cycle ^15^ we discovered a pathway dealing with 2-phosphoglycolate, the side product of RuBisCO oxygenation reaction^18^. Such findings were the motivation behind our search for other undiscovered pathways that focus on methanol, formate, and CO_2_ assimilation routes that are naturally active.

**Fig. 1:**
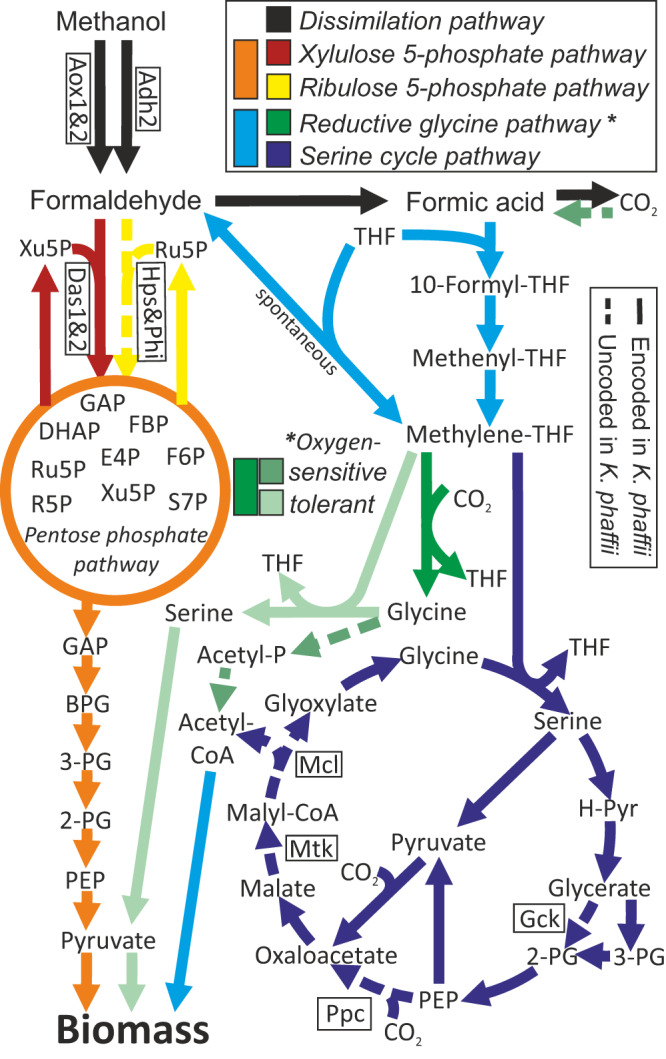
Scheme describing methanol and formate assimilation pathways. The natural main methanol assimilation pathway in *K. phaffii*, the xylulose 5-phosphate pathway, and the ribulose 5-phosphate pathway (*B. methanolicus*) are methanol assimilation pathways that fix formaldehyde. Glyceraldehyde phosphate is produced via pentose phosphate pathway reactions and is then the key metabolite for all biomass production. As methanol is dissimilated, the reductive glycine pathway and the serine cycle are methanol and formate assimilation pathways. Formate fixation occurs in the tetrahydrofolate cycle, and both co-assimilate CO_2_. The O_2_-tolerant reductive glycine pathway leads to pyruvate, while the O_2_-sensitive reductive glycine pathway (*D. desulfuricans*) and the serine cycle (*M. extroquens*) culminate in acetyl-CoA as a subsequent metabolite for biomass production. Reactions focus on those involving carbon only. For the other compounds, enzymes and gene names involved, see Supplementary Fig. 1 & 2. Dashed reactions are not encoded in *K. phaffii*. Abbreviations: THF tetrahydrofolate, R5P ribose 5-phosphate, S7P sedoheptulose 7-phosphate, E4P erythrose 4-phosphate, Xu5P xylulose 5-phosphate, Ru5P ribulose 5-phosphate, FBP fructose bis-phosphate, DHAP dihydroxyacetone phosphate, GAP glyceraldehyde phosphate, BPG bis-phosphoglycerate, 2- & 3-PG 2-& 3-phosphoglycerate, PEP phosphoenolpyruvate, H-Pyr hydroxyl-pyruvate, Acetyl-P acetyl-phosphate, Adh alcohol dehydrogenase, Aox alcohol oxidase, Das dihydroxyacetone synthase, Hps 3-hexulose-6-phosphate synthase, Phi phosphohexose isomerase, Gck glycerate 2-kinase, Pdc phosphoenolpyruvate carboxylase, Mtk malate-CoA ligase, Mcl malyl-CoA lyase.

In nature, several methanol fixation pathways are present and functional. These are differentiated into formaldehyde- and formate-fixation pathways (see Fig. 1 and Supplementary Fig. 1 & 2). Similar to XuMP, the ribulose 5-phosphate (RuMP) pathway which is present in many methylotrophic bacteria, e.g. in *Bacillus methanolicus*, is a formaldehyde-fixation pathway (Fig. 1, red & orange). Here, the pentose phosphate pathway (PPP) is used to recycle a sugar phosphate and produce glyceraldehyde 3-phosphate (GAP) that is subsequently used in biomass formation^19,20^. As shown in engineered *Escherichia coli* that utilise formaldehyde^21^ and methanol^22,23^ for growth, the enzymes 3-hexulose 6-phosphate synthase (Hps) and phosphohexose isomerase (Phi) are of central importance in RuMP. After adaptive laboratory evolution, *Saccharomyces cerevisae* could also grow on methanol via a formaldehyde-fixation pathway that involves the PPP^24^. In the serine cycle pathway of e.g. *Methylorubrum extorquens* (formerly *Methylobacterium extorquens*^25^), methanol is dissimilated to formaldehyde and then to formate via a formate-fixation pathway. Subsequently, formate enters the enzymatic tetrahydrofolate (THF) pathway leading to methylene-THF^26,27^. In addition to this formate-fixation pathway, to a minor extent, it may also fixate formaldehyde because of the spontaneous in vivo condensation reaction of THF with formaldehyde-forming methylene-THF (see Fig. 1, cyan)^26,28^. In the serine cycle, the carbon originating from methanol or formate is condensed with a second carbon from CO_2_ culminating in acetyl-CoA that is then used in all biomass formation (see Fig. 1, blue reactions in the cycle)^26,29^. Therefore, the serine cycle is a CO_2_-fixation pathway. An engineered version of the serine cycle via pyruvate that follows an alternative CO_2_-fixation reaction has been demonstrated in *E. coli* (see Fig. 1, linear blue reactions)^30^. Another synthetic route engineered in *E. coli* is the homoserine cycle, which captures two aliquots of methanol to produce acetyl-CoA via a cyclic regeneration of glycine via homoserine and threonine^31^.

The oxygen-sensitive reductive glycine pathway is the native CO_2_ fixation pathway of the anaerobic bacterium *Desulfovibrio desulfuricans*^32^. CO_2_ is reduced to formate by an oxygen-sensitive enzyme, formate is consecutively metabolized to methylene-THF via the tetrahydrofolate pathway. In the reaction defining the general reductive glycine pathway, a second CO_2_ molecule is incorporated to the de novo synthesised amino acid glycine via the glycine cleavage system. Glycine is metabolised to acetyl-phosphate by another oxygen-sensitive reaction before culminating in acetyl-CoA that can either be used directly for biomass production or partially incorporates a third carbon dioxide molecule to form pyruvate^32^. In the acetogenic bacterium *Clostridium drakei* the reductive glycine pathway cooperates with the Wood-Ljungdahl pathway for autotrophic growth^33^. The reductive glycine pathway from formate to glycine was overexpressed in a glycine auxotrophic *S. cerevisiae* strain and led to growth on glucose, formate, and CO_2_^34^. The reversibility of the glycine cleavage system in yeast was thus proven. The following reaction from glycine to serine in the reductive glycine pathway was recently demonstrated as well with a serine auxotrophic yeast strain, where compensation for the serine auxotrophy via this pathway with formate and CO_2_ could only be achieved with an overexpressed heterologous gene^35^. The enzymes catalysing the conversion of formate to pyruvate are encoded in the yeast genome so that the pathway into the central carbon metabolism should be possible although growth without glucose could not be demonstrated in this work. This so-called oxygen-tolerant reductive glycine pathway is known from metagenomic analyses as a purely CO_2_-fixation route^36^. It has also been designed and integrated as a synthetic pathway in *E. coli* where it supported growth on formate and methanol in combination with CO_2_^37–39^. The Calvin cycle in *Cupriavidus necator* was successfully exchanged by the synthetic reductive glycine pathway^40^ and recently the same pathway was partially integrated and tested via auxotrophies in *Pseudomonas putida*^41,42^. Nevertheless, proof of innate metabolic activity of the oxygen-tolerant reductive glycine pathway has not yet been observed in nature for any organism.

In this study, employing metabolic tracer analysis with ^13^C-labelled methanol in a *K. phaffii* XuMP knockout strain (*das1Δdas2Δ*), we find that there is an alternative route of methanol assimilation in yeast, and identify it as a variant of the reductive glycine pathway. GC-TOFMS methods^43^ allow for the verification of the carbon transition from methanol via methylene-THF plus CO_2_ to glycine, and from glycine and methylene-THF to serine, from where the carbon enters the central metabolism. We show that formate is assimilated via the same route. Deletion of the mitochondrial isogene of serine hydroxymethyltransferase (*SHM1*) relieves the competition of this enzyme with the mitochondrial glycine cleavage system for methylene-THF and enables a strain to assimilate methanol or formate, respectively, together with CO_2_ at a rate that is fast enough to sustain growth and cell division.

Our work points out that such naturally encoded pathways for methanol or formate co-assimilation with CO_2_ are present in eukaryotes as well and can be awakened by metabolic engineering, and form a basis for sustainable single-carbon bioprocesses with industrially-relevant yeasts in the future.

## Results

### ^13^C-methanol labelling indicates the presence of the reductive glycine pathway *in K. phaffii*

**Fig. 2:**
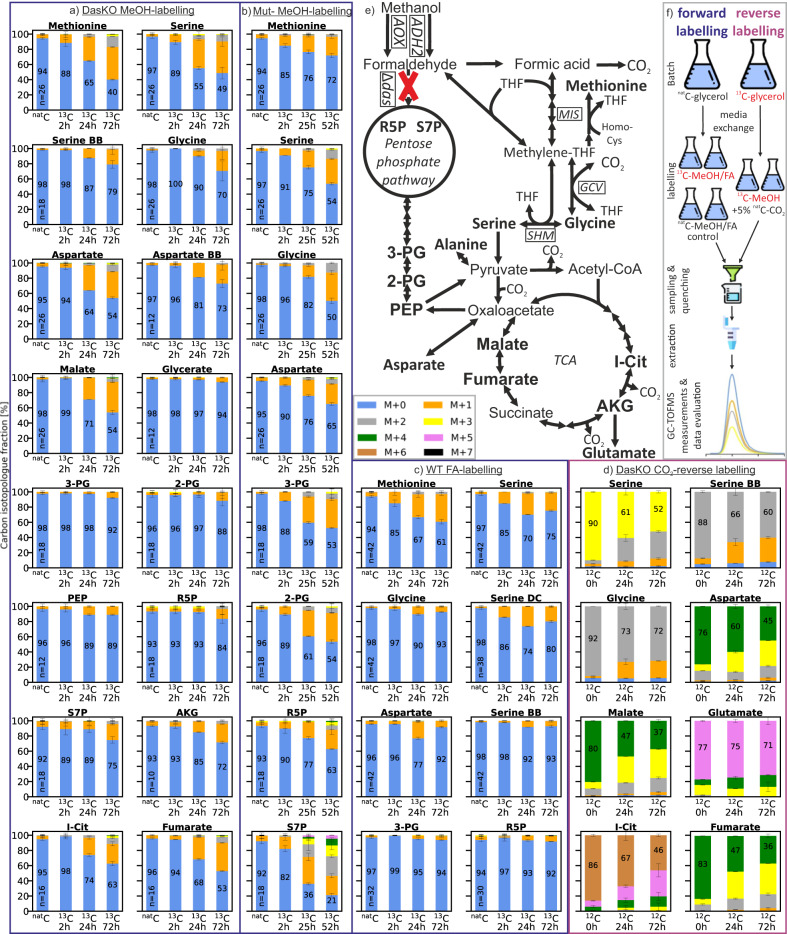
Carbon isotopologue distribution analysis via GC-CI/EI-TOFMS. **a**–**d** *K. phaffii* strains (see Table 1) labelled with different carbon sources (*n* = 2 biological replicates for labelled strains, number of replicates (n) of the ^nat^C controls is indicated in the bar). “BB” in the metabolite name refers to the amino acid backbone, i.e., C1 and C2 only; “DC” refers to the decarboxylated amino acid, *i.e*., all carbon atoms excluding C1 (see Supplementary Data 3), the molecular structures of these fragments are shown in Supplementary Fig. 3 and for serine as an example in Fig. 3f. The number x in”M + x” indicates the number of ^13^C-carbon atoms and thus specifies the respective isotopologue. As shown in **a**–**c** for the forward labelling experiments, pathway routes were assessed by tracing the relative ^13^C abundance in the metabolites. Generally, an upstream metabolite of any active pathway must contain more ^13^C than the corresponding downstream metabolite. An increase in ^13^C-content resulted in a decrease in the isotopologue M + 0 that contains ^12^C only. For all forward labelling approaches, the isotopologue fraction of M + 0 is also indicated as a number in the corresponding bar, see (**a**–**c**). For reverse labelling (2d), ^12^C incorporation was traced. Therefore, any decrease in abundance of isotopologues containing ^13^C indicated CO_2_ incorporation. (**a**) *das1Δdas2Δ* strain labelled with ^13^C-methanol for 72 h without any other carbon source; (**b**) *aox1Δaox2Δ* strain labelled with ^13^C-methanol for 52 h without any other carbon source; (**c**) wildtype strain labelled with ^13^C-sodium formate for 72 h without any other carbon source; (**d**) ^13^C-labeled *das1Δdas2Δ* strain reverse labelled with 5% ^nat^C-CO_2_ (^12^C tracing) and fed with ^13^C-methanol; (**e**) reductive glycine pathway and native xylulose 5-phosphate pathway illustrated to the TCA cycle, all measured metabolites are highlighted with bold letters; (**f**) illustration of labelling and reverse labelling workflow. Labelling data of additional metabolites, see Supplementary Fig. 7; Abbreviations: MeOH methanol, FA formic acid, THF tetrahydrofolate, TCA tricarboxylic acid cycle, R5P ribose 5-phosphate, S7P sedoheptulose 7-phosphate, 2-PG 2-phosphoglycerate, 3-PG 3-phosphoglycerate, PEP phosphoenolpyruvate, AKG α-ketoglutarate, I-Cit isocitrate. Error bars in **a**–**d** represent corrected standard deviations of mean values. Icons in (**f**) were obtained from Freepik (Fuels) at https://www.flaticon.com/. Source data are provided as a Source Data file.

The XuMP knockout strain (DasKO; for strain genotypes see Table 1) does not grow on methanol alone. However, when cultivated on ^13^C-methanol without any other carbon source, the presence of an alternative methanol assimilation pathway became apparent as observed by a temporal increase in the ^13^C content of various metabolites (Fig. 2a). As shown in Fig. 2a-c, the pathway routes were assessed by tracing the relative ^13^C abundance in the metabolites. In these figures, the number x of ^13^C-atoms in a metabolite is denoted by “M + x´´, thus specifying the respective isotopologue. An increase in ^13^C-content is always associated with a decrease in the M + 0 (^12^C only) isotopologue fraction. This M + 0 fraction is also indicated as a numerical value in the corresponding bar for all forward labelling approaches. Generally, an upstream metabolite of any active pathway contains a higher fraction of ^13^C than the corresponding downstream metabolites. Further parameters like metabolite pool sizes, reaction rates and compartmentalisation of reactions need to be considered as well. Compounds that have the fastest and highest incorporation of the ^13^C label are indicative at the beginning of an active inherent pathway.

**Table 1:** Names and genotypes of strains used in this study

Strain name	Genotype	Source
WT	CBS7435	
DasKO	CBS7435 *das1Δdas2Δ*	This study
GcvKO	CBS7435 *das1Δdas2Δ gcv1Δgcv2Δ*	This study
Shm1KO	CBS7435 *das1Δdas2Δ shm1Δ*	This study
ShmKO	CBS7435 *das1Δdas2Δ shm1Δshm2Δ*	This study
MisKO	CBS7435 *das1Δdas2Δ mis1-1Δmis1-2&3Δ::loxP-kanMX-loxP*	This study
GcvOE	CBS7435 *das1Δdas2Δ P*_*FDH1*_*GCV1 P*_*DAS2*_*GCV2 P*_*AOX1*_*LPD1 P*_*DAS1*_*GCV3*	This study
OtRedGlyOE	CBS7435 *das1Δdas2Δ P*_*FDH1*_*GCV1 P*_*DAS2*_*GCV2 P*_*AOX1*_*LPD1 P*_*DAS1*_*GCV3 P*_*DAS1*_*MIS1-1 P*_*AOX1*_*SHM1 P*_*DAS2*_*CHA1 P*_*DAS1*_*ADE3 P*_*AOX1*_*SHM2*	This study
Shm1KO MeMisOE	CBS7435 *das1Δdas2Δ shm1Δ P*_*DAS1*_*Fhs P*_*CS1*_*FchA P*_*TEF1*_*MtdA*	This study
Mut^-^	CBS2612 *aox1Δaox2Δ*	Zavec et al. ^17^
Single MisKO	CBS7435 *Δdas1Δdas2 Δmis1-1*	This study
mitoOtRedGlyOE	CBS7435 *Δdas1Δdas2 P*_*FDH1*_*GCV1 P*_*DAS2*_*GCV2 P*_*AOX1*_*LPD1 P*_*DAS1*_*GCV3 P*_*DAS1*_*MIS1-1 P*_*AOX1*_*SHM1 P*_*DAS2*_*CHA1*	This study
Das&Aox1KO	CBS7435 *das1Δdas2Δ aox1Δ*	Gassler et al. ^15^

Of all the metabolites measured, methionine and serine showed a strong decrease of 6-8% in the unlabelled M + 0 fraction after only 2 hours post labelling; and the highest label incorporation at all three of the time points (2, 24 and 72 h). Alternative to the XuMP pathway, these data clearly showed that methanol is primarily assimilated via the tetrahydrofolate pathway because methionine and serine are produced from this pathway. Serine showed an increase in the isotopologue M + 2 after 24 h. This indicated that a second labelled carbon atom originating from methylene-THF is incorporated via glycine. Glycine increased in ^13^C content 24 hours post labelling but less than serine, which indicates a lower activity of the glycine cleavage system compared to the serine hydroxymethyl transferase. The mass spectrometric fragment ions from serine and aspartate are specific for the amino acid backbone (BB) as these contained the C1 and C2 carbons of the amino acids only (see Supplementary Fig. 3). Both showed a very similar labelling pattern to glycine. The data further supports the involvement of glycine and the enzyme serine hydroxymethyltransferase (Shm) in the alternative methanol pathway. After 24 hours, an increase in the ^13^C content of aspartate and malate was also observed. Malate and aspartate are direct downstream metabolites of oxaloacetate that is produced by carboxylation of pyruvate. The appearance of ^13^C in malate and aspartate is therefore an initial evidence that serine is first deaminated to pyruvate to subsequently act as the labelled precursor. If labels in these two metabolites would derive from the oxidative TCA cycle, more label incorporation is expected in isocitrate (I-Cit), α-ketoglutarate (AKG) and glutamate. The labelling pattern of oxaloacetate was not determined because the concentration in the extracts was below the limit of detection of the method. Pyruvate was observed as a degradation product of both oxaloacetate and phosphoenolpyruvate (PEP). These metabolites originate from two different pathways, thus pyruvate could not be evaluated. Generally, such metabolic interconversion reactions (non-enzymatic in vitro reactions) can falsify results and consequently, pathway interpretation. Therefore, critical analytes of the hypothesised pathways were assessed (detailed information in Supplementary Note 1 and Supplementary Fig. 4-6). Alanine may be used as a proxy for pyruvate labelling. Its ^13^C pattern was very similar to PEP and 2&3-PG and lower than serine (Supplementary Fig. 7a), indicating that a feedback from PEP via pyruvate to alanine makes it difficult to draw conclusions from alanine labelling on the pathway.

If formaldehyde fixation pathways acting through the pentose phosphate pathway (PPP) and glycolysis would be active, then a different pattern would be expected. Namely, metabolites of these pathways, *e.g*., sedoheptulose 7-phosphate (S7P), ribose 5-phosphate (R5P), 2&3-PG would show an earlier and higher degree of ^13^C labelling than methionine, serine and glycine, as well as the downstream metabolites aspartate, malate and fumarate. Labelling data of wild type *K. phaffii* cannot directly be compared with the *das1Δdas2Δ* strain used here, as the growing cells incorporate ^13^C much faster. Therefore, we used an *aox1Δaox2Δ* (methanol utilisation negative or Mut^-^) strain as a control that still dissimilates methanol to formaldehyde via *ADH2*^17^ and has the native xylulose 5-phosphate pathway but does not grow on methanol. The labelling patterns of metabolites in this control strain matches the expected patterns described above (Fig. 2b). The low degree of ^13^C-labels observed in the glycolysis and pentose phosphate pathway metabolites in the DasKO strain can be explained by the incorporation of ^13^C from oxaloacetate via gluconeogenesis. The native serine cycle route via glycerate, 2-PG and 3-PG to oxaloacetate (see Fig. 1) is obviously not active in *K. phaffii* as malate and aspartate showed higher labelling values than 2-PG, 3-PG and PEP. Still, as reflected in the labelling data, the reactions of the modified serine cycle of Yu and Liao^30^ via pyruvate to malate were active. If the reactions resulting in acetyl-CoA are active, a higher ^13^C content in the tricarboxylic acid (TCA) cycle metabolites isocitrate (I-Cit) and α-ketoglutarate (AKG) would be expected. These are the first metabolites that can be measured after the acetyl-CoA fixation point in the TCA cycle.

When additional ^nat^C glycine was supplied to the medium and the methanol labelling was performed, the same labelling pattern was observed although the intensity of the labels was reduced. This is a consequence of the reaction of glycine to methylene-THF resulting in a mixed feed of methanol and glycine; and additionally provides evidence for the general activity of the glycine cleavage system (see Supplementary Fig. 7e; labelling results for more metabolites than shown in Fig. 2a & b are given in Supplementary Fig. 7a & b).

Briefly, it can be concluded from the ^13^C methanol labelling data obtained from the DasKO strain that the oxygen-tolerant reductive glycine pathway from methanol to oxaloacetate is active in vivo in *K. phaffii*, however not to an extent that it can support growth.

### ^13^C-formate labelling demonstrates formate assimilation via the reductive glycine pathway

Formate (FA) is an intermediate metabolite of methanol dissimilation. If the oxygen-tolerant reductive glycine pathway is the alternative methanol assimilation pathway in *K. phaffii*, formate can also be used as a carbon source for this organism via the same pathway. Hence, a ^13^C-formate tracer experiment was designed and performed to further validate the activity of the pathway. The result should provide evidence on whether formate is assimilated via the enzymatic tetrahydrofolate pathway or whether methylene-THF is generated directly via the spontaneous reaction of THF and formaldehyde. As depicted in Fig. 2c, the formate labelling data of the wildtype strain showed a comparable labelling pattern to the methanol labelling data (Fig. 2a) at 2 and 24 hours. The earliest and most substantial labelling occurred for methionine and serine. Glycine, aspartate and the serine backbone (reflecting the carbon atoms in serine that originate from glycine) were significantly labelled at the second time point (24 h); and were labelled to a higher degree than the metabolites of glycolysis (3-PG) and the pentose phosphate pathway (R5P). Excluding methionine, at 72 hours the degree of labelling was decreased for all analysed metabolites. As shown from the analysis of the culture medium (see Supplementary Fig. 8), this is consistent with the reduced consumption of formate after 24 hours of cultivation.

Formate dissimilation provides only half the energy of methanol (one NADH compared to two molecules for methanol) which obviously leads to severe starvation after 24 hours of cultivation. A limitation of supply with ATP and reducing equivalents impairs further assimilation of formate into methylene-THF so that no further ^13^C label would be incorporated at this stage. The pool of unlabelled metabolites of the biomass is a likely source of ^12^C, leading to the lower degree of labelling at 72 hours compared to 24 hours. Compared to the wildtype strain, formate-labelling of the DasKO strain resulted in similar patterns at all time points and for all metabolites (see Supplementary Fig. 7f). Hence, *K. phaffii* can natively assimilate formate through the oxygen-tolerant reductive glycine pathway. A reverse reaction from formate to formaldehyde followed by diffusion in the peroxisome and fixation via the xylulose 5-phosphate pathway could not be proven with this method as the wildtype and the DasKO showed similar labelling patterns. Furthermore, for all experiments, the data indicated that ^13^C labels in the pentose phosphate pathway and in glycolysis are derived from gluconeogenesis initiated from oxaloacetate. Thus, our postulation from the previous section was confirmed (labelling data of additional metabolites: Supplementary Fig. 7c).

### CO_2_ reverse labelling proves that methanol and CO_2_ are co-assimilated to pyruvate and further to oxaloacetate

To trace the co-assimilation of CO_2_ via the reductive glycine pathway, or more specifically, via the glycine cleavage system, a CO_2_ reverse labelling experiment was performed. By avoiding the use of ^13^C-CO_2_, the DasKO strain was fully labelled with ^13^C-glycerol followed by reverse labelling with ^nat^C-CO_2_ under ^13^C-methanol addition (see Fig. 2). For reverse labelling, ^12^C incorporation was traced. Therefore, any decrease in abundance of isotopologues containing ^13^C indicated incorporation of CO_2_. The experiment clearly revealed that native CO_2_-fixation pathways are active because the ^13^C content of the metabolites decreased during the cultivation time, *i.e*., the isotopologue distribution pattern was shifted towards isotopologues with a lower number of ^13^C atoms (Fig. 2d). Serine and glycine were also reverse labelled, thus providing further evidence of the activity of the anabolically-acting glycine cleavage system that results in CO_2_-fixation. A second carboxylation reaction in this route, *i.e*., pyruvate to oxaloacetate, was verified by the fact that aspartate and malate showed the most intense reverse labelling. The temporal increase in the isotopologues M + 3 and M + 2 was indicative of double CO_2_-fixation through the reductive glycine pathway to oxaloacetate. This occurred via carboxylation through the glycine cleavage system and via carboxylation by pyruvate carboxylase.

### The genome of *K. phaffii* encodes all enzymes of the reductive glycine pathway

The xylulose 5-phosphate pathway (XuMP) is the most active methanol assimilation pathway in *K. phaffii* and has been studied in detail^16^. To identify alternative methanol assimilation pathways in silico, the native formaldehyde fixing dihydroxyacetone synthase (*DAS1, DAS2*) was firstly considered deleted. The natively-encoded enzymes and resultant pathways for the assimilation of methanol were then compared to the pathways of other methylotrophic organisms. For the ribulose 5-phosphate pathway (RuMP), only 3-hexulose-6-phosphate synthase (Hps) and phosphohexose isomerase (Phi) are absent in *K. phaffii* (Fig. 1, yellow dotted reaction). Still, native but unknown formaldehyde-condensing aldolase activities in *K. phaffii*^44^ need to be taken into consideration, *e.g*., the evolved *S. cerevisae* strain mentioned in the introduction^24^. In *K. phaffii*, all formate assimilation pathways are also methanol assimilation pathways because formate is produced from the dissimilation pathway of methanol (Fig. 1, black reactions). For the native serine cycle, which is a formate assimilation pathway, four enzymes are not encoded by *K. phaffii* (Fig. 1, dark blue dotted reactions). If a shortcut via pyruvate is considered, malate-CoA ligase (Mtk) and malyl-CoA lyase (Mcl) are the only enzymes not encoded for acetyl-CoA synthesis. As suggested, all the other necessary enzymes from methanol, or formate, to malate are encoded and active according to the labelling experiments (Supplementary Fig. 2).

To discover potential additional CO_2_ assimilation pathways in silico, a search for enzymes involved in CO_2_ fixation and pathways inspired by organisms that grow on CO_2_ as the sole carbon source was initiated. As observed in the oxygen-sensitive reductive glycine pathway of *D. desulfuricans*, the oxygen-sensitive glycine reductase complex to acetyl-phosphate and the phosphate acetyltransferase, or the acetate kinase, to acetyl-CoA are not encoded in the genome of *K. phaffii* (see Fig. 1, light green dotted reactions). Other native or synthetic methanol or formate assimilation pathways are improbable either due to the requirement for anaerobic conditions, *e.g*., the reductive acetyl-CoA pathway, or due to the absence of several genes related to pathway enzymes. This is the case for the homoserine cycle^31^, the serine-threonine cycle and other synthetic routes proposed by Bar-Even in 2016^45,46^. The oxygen-tolerant reductive glycine pathway is the only pathway where all enzymes were encoded (see Fig. 1, green reactions to pyruvate; details in Supplementary Fig. 1). This bioinformatic analysis confirmed the results of the labelling experiment and strengthened the hypothesis that the oxygen-tolerant reductive glycine pathway is a native, metabolically-active methanol, formate and CO_2_ assimilation pathway in *K. phaffii*.

During genome mining, we discovered that the cytosolic folate pathway gene MIS (encoding C1 tetrahydrofolate synthase) was split into two genes. Subsequently, there is a higher expression of the formate-tetrahydrofolate ligase subunit compared to the methenyltetrahydrofolate cyclohydrolase and dehydrogenase subunits, based on transcriptome data^47^ (Supplementary Fig. 9-11). This split may result in a higher flux towards 10-formyl-THF formation on methanol, which is required for purine de novo synthesis (see Supplementary Note 2).

### Additional knockout and overexpression strains confirm the presence of an active reductive glycine pathway

**Fig. 3:**
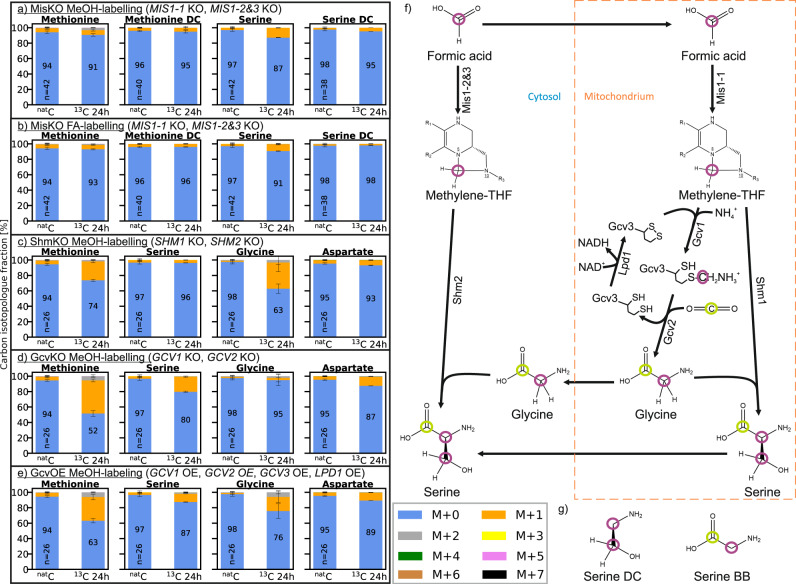
Carbon isotopologue distribution analysis of additional knockout strains. As in Fig. 2, *K. phaffii* strains (see Table 1) are labelled with different carbon sources (*n* = 2 biological replicates for labelled strains, number of replicates (*n*) of the ^nat^C controls is indicated in the bar). “BB” in the metabolite name refers to the amino acid backbone, i.e., C1 and C2 only, “DC” refers to the decarboxylated amino acid, i.e., all carbon atoms except C1 (molecular structure of serine see (**f**)). (**a**) *das1Δdas2 Δmis1-1Δmis1-2&*3*Δ* strain labelled with ^13^C-methanol for 24 h; (**b**) *das1Δdas2Δ mis1-1Δmis1-2&3Δ* strain labelled with ^13^C-sodium formate for 24 h; (**c**) *das1Δdas2Δ shm1Δshm2Δ* strain labelled with ^13^C-methanol for 24 h; (**d**) *das1Δdas2Δ gcv1Δgcv2Δ* strain labelled with ^13^C-methanol for 24 h; (**e**) *das1Δdas2Δ* P_strong_*GCV1&2&3&LPD1* strain labelled with ^13^C-methanol for 24 h; (**f**) the reductive glycine pathway with molecular structures, compartments and all overexpressed or deleted genes. Carbon derived from methanol or formate is marked with a purple circle, carbon from CO_2_ with a green circle; (**g**) structures of the fragments of serine “BB” and “DC” as described above, for derivatized structures see Supplementary Fig. 3. Labelling data of additional metabolites, see Supplementary Fig. 7 & 12. Error bars in a-d represent corrected standard deviations of mean values. Source data are provided as a Source Data file.

To explore if the reductive glycine pathway is the only alternative methanol and formate assimilation pathway, further targeted gene knockouts were conducted (summarised in Table 1) and assessed in forward labelling experiments. The enzymatic formate fixation pathway to methylene-THF was disrupted by deleting the *MIS* genes (MisKO strain). Formate- and methanol-labelling experiments with this strain led to the following result. After 24 h, the fraction of unlabelled isotopologue M + 0 from methionine and serine was only slightly reduced indicating that a minor amount of ^13^C-label was incorporated (Fig. 3a & b). In comparison, the Mis active DasKO strains were heavily-labelled at the same time point. The decarboxylated (DC) mass spectrometric fragments of these amino acids (see Supplementary Fig. 3), that only contain carbon atoms derived from methanol or formate in the proposed pathway, did not show any increase in ^13^C content. This is in agreement with the underlying hypothesis. The unfragmented amino acids are labelled with ^13^C to a minor degree because the label is located in the carboxy groups of the amino acids and these were derived from the active carboxylation of the glycine cleavage system (Gcv). Due to the fact that the dissimilation pathway to CO_2_ is still active, ^13^C-CO_2_ is produced intracellularly and re-fixation via the Gcv system can occur. Consequently, the labels detected in the unfragmented amino acids originate from the refixation of ^13^C-CO_2_, and not from the direct fixation of formate or methanol. To remove any doubt as to whether the labelling patterns derived from the experiments with the DasKO strain and the wildtype (described in the prior sections) stem from intracellular ^13^C-CO_2_, the decarboxylated amino acid fragments from these experiments were also evaluated (Fig. 3 and Supplementary Fig. 7 & 12). As these were ^13^C-labelled, the carbon atom must have indeed originated from formate. This implies that no other formate or methanol incorporation pathway was found. The spontaneous in vivo condensation of THF and formaldehyde to methylene-THF was not observed when cultivating the MisKO strain on methanol; hence, it is not present in the cytosol to an extent that is detectable with the used GC-TOFMS methods. When the *SHM* genes were deleted, the direct downstream metabolite serine remained unlabelled. Upstream methionine and glycine, however, were still labelled (Fig. 3 c). This is in accordance with the underlying hypothesis and confirmed that the labels in glycine do not just originate from serine via Shm enzymes.

For all experiments, glycine was labelled later, and to a lesser degree, than serine. Thus, it is not unreasonable to assume that glycine is downstream of serine. This suggestion raised doubts as to whether glycine is indeed upstream of serine with the labels originating from the glycine cleavage system (Fig. 3f) and entering the detected pathway. To underline the involvement of the gcv system and ascertain if glycine is actually upstream of serine, the gcv system was both deleted and overexpressed. In the GcvKO strain, no significant incorporation of ^13^C into glycine was observed (Fig. 3d) which led to the conclusion that the labels in glycine are only incorporated via the glycine cleavage system. As serine is downstream in the proposed pathway and can incorporate labels from only one of the two reactions, the ^13^C amount is also less than that observed in the DasKO strain. When gcv was overexpressed, increased incorporation of ^13^C was evident. More precisely, a reduction of the unlabelled isotopologue M + 0 from 90% to 75% after 24 hours was apparent (Fig. 3e). Labelling data of further metabolites are given in the Supplementary Fig. 12.

### Tuning the native reductive glycine pathway enables mixotrophic growth on methanol or formate and CO_2_

**Fig. 4:**
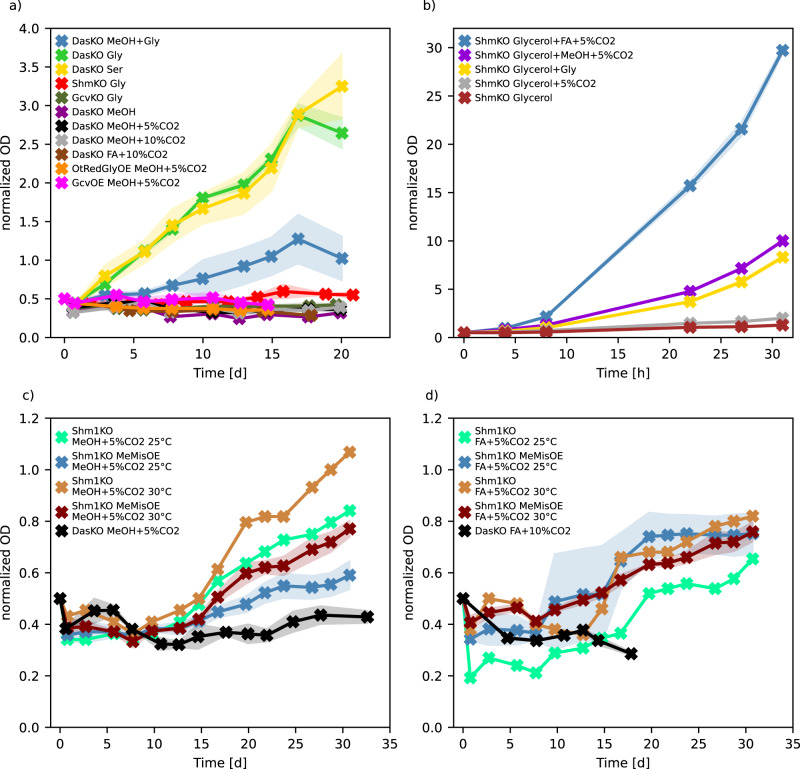
Results of growth analysis. Cultures were inoculated at an OD_600_ of approximately 0.5. To aid comparison, all data was normalised to an initial value of 0.5. All strains assessed have *DAS1&2* knockouts, some strains have additional knockouts or overexpressed genes as indicated (genotypes are listed in Table 1). Data are average values from biological duplicates with the standard deviation indicated by the shaded area. Long-term cultivation (up to 46 days), repeatability studies, non-normalised data and further cultivation of overexpressed strains are shown in Supplementary Fig. 13 & 14. (**a**) *DAS1&2* knockout strains cultivated on methanol or formate, with elevated CO_2_, or the addition of glycine or serine, and the overexpression of the reductive glycine pathway. (**b**) SHM double knockout strains grown on glycerol with supplementation of methanol, formate and CO_2_, as indicated, and of glycine as a control. (**c**) *SHM1* knockout strain both with and without overexpression of *M. extorquens* MIS genes, grown with methanol and CO_2_ at two different temperatures, and the parental DasKO strain as a negative control. (**d**) Same strains as in Fig. 4c grown on formate and CO_2_. Since cultures started to flocculate in cultivation of Shm1KO and Shm1KO MeMisOE on formate additional cell counting was performed to verify growth. Data is shown in Supplementary Fig. 14c for cultures on methanol (as a non-flocculating control) and in Supplementary Fig. 14d for cultures on formate. Source data are provided as a Source Data file.

The xylulose 5-phosphate pathway knockout strain (DasKO) cannot grow on methanol as the only carbon source (Fig. 4a). Nevertheless, we demonstrated that methanol and formate are assimilated in this strain, while co-assimilating CO_2_. Therefore, we tested for growth on methanol and formate with elevated CO_2_ levels of 5% or 10%, however, still no growth was observed. The cells consumed these carbon sources and formate was even secreted when methanol was dissimilated (see Supplementary Fig. 8). As formate is produced intracellularly via the methanol dissimilation pathway and so is available to the cell, most conditions were only assessed on methanol. Insufficient expression of genes involved in the reductive glycine pathway was not an obvious limitation, as neither overexpression of parts or the entire pathway induced growth on mixotrophic conditions (Fig. 4a and Supplementary Fig. 13).

**Fig. 5:**
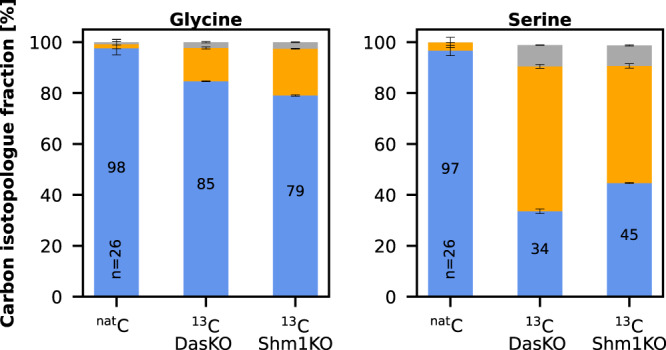
Carbon isotopologue distribution analysis of growing Shm1KO strain. *K. phaffii* strains *das1Δdas2 shm1Δ* and *das1Δdas2* as control were labelled with ^13^C-methanol for 24 h with 5% CO_2_ (with natural isotope distribution) added in the atmosphere. Data display is the same as in Fig. 2 & 3 (*n* = 3 biological replicates for labelled strains, number of replicates (*n*) of the ^nat^C controls is indicated in the bar). Error bars represent corrected standard deviations of mean values. Source data are provided as a Source Data file.

**Fig. 6:**
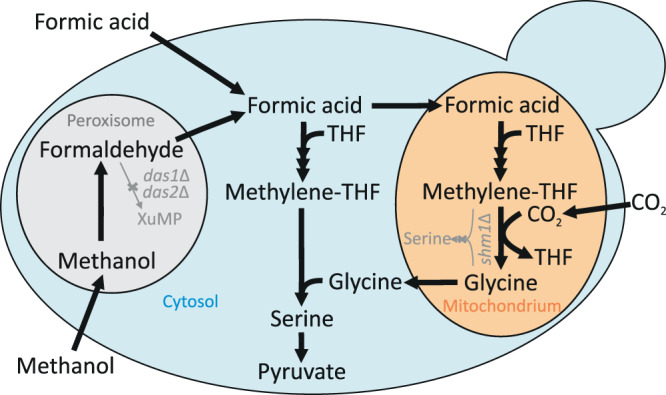
The growth supporting oxygen-tolerant reductive glycine pathway in *K. phaffii*. The oxygen-tolerant reductive glycine pathway is the sole, natively-active alternative methanol assimilation pathway in the yeast *K. phaffii*; and is also the sole, native formate and native CO_2_ assimilation pathway. It is initiated via methanol or formate assimilation and continues via methylene-THF, glycine and serine towards the formation of pyruvate and even further towards the formation of oxaloacetate. With the deletion of *SHM1 DAS1&2* double knockout strains can grow via the displayed and compartmentalized pathway without any overexpressions. Abbreviations: THF tetrahydrofolate; TCA tricarboxylic acid cycle; XuMP xylulose 5-phosphate pathway; PPP pentose phosphate pathway.

The addition of glycine to DasKO cultures enabled growth without a further carbon source (Fig. 4a), therefore the downstream pathway from glycine to pyruvate into the central carbon metabolism must be active. Deletion of both SHM genes, on the other hand, creates a glycine limitation during growth on e.g. glycerol, which is overcome by the addition of 1% methanol and 5% CO_2_ which was even outperformed when exchanging methanol with 30 mM formate (Fig. 4b). This indicates that the synthesis of glycine via the reverse glycine cleavage system is markedly enhanced when the competition for methylene-THF between GCV and SHM is abolished. We have consequently designed a DasKO strain with a deletion of *SHM1* (encoding the mitochondrial version of the enzyme) to enable more of the carbon flux from methylene-THF to glycine, while serine synthesis is still enabled by cytosolic Shm2. This strain can grow on methanol and formate, respectively, both with elevated CO_2_ concentrations, but without addition of any further carbon source (Fig. 4c & d and Supplementary Fig. 14). Growth data were confirmed by labelling with ^13^C methanol. After 24 hours ^13^C was elevated in glycine in the Shm1KO strain and lowered in serine (Fig. 5) in comparison to the parental DasKO strain, supporting the hypothesis that the reverse glycine cleavage reaction is limited by competition with Shm1 for mitochondrial CH_2_-THF. Labelling with ^13^C formate was also attempted, however, due to the obvious energy limitation of these cultures the data were not reproducible. We conclude that the growth limitation could be overcome by making use of the native compartmentalisation of the pathway by producing glycine from formate in mitochondria, avoiding the drain of CH_2_-THF to serine with the *SHM1* knockout in the same compartment. Glycine is then transported to the cytosol where the cytosolic Shm2 transfers carbon coming from formate via the cytosolic THF-pathway onto glycine-producing serine, which is deaminated in the same compartment to pyruvate which is then the main precursor for all biomass formation (see Fig. 6). Co-overexpression of *M. extorquens* Mis genes (*Fhs, FchA, MtdA*) had no positive effect on growth rate, indicating that the main limit is the flux competition at the GCV/SHM node.

## Discussion

Carbon metabolism enzymes are generally amongst the most abundant cellular proteins so that high flux rates are enabled. On any given substrate, the most abundant catabolic pathway typically dominates and less active pathways are easily neglected in biochemical analyses. In *K. phaffii*, the canonical methanol assimilation pathway is initiated by alcohol oxidase (Aox) to formaldehyde and enters the XuMP cycle to generate glyceraldehyde 3-phosphate. Recently, we showed that Aox is not the only enzyme that catalyses the first reaction step in native methanol metabolism. Known for both synthesis and consumption of ethanol, the cytosolic alcohol dehydrogenase (Adh2) also oxidises methanol to formaldehyde^17^. This reaction conserves energy by the reduction of NAD^+^ to NADH; but there appears to be some limitations because with Adh only, growth was not observed. Here, we demonstrate that *K. phaffii* harbours a complete methanol, or formate and CO_2_ co-assimilation pathway that firstly leads to pyruvate and then to oxaloacetate. This oxygen-tolerant reductive glycine pathway provides precursors for all metabolic routes in yeast (see Fig. 6).

Although the native metabolic activity of the oxygen-tolerant reductive glycine pathway from formate or methanol to pyruvate or oxaloacetate, respectively, has not been observed in nature it is not completely unknown. The pathway has been reported on a metagenomic level for the anaerobic bacterium *Candidatus Phositivorax anaerolimi Phox-21*^36^. This organism, however, has yet to be isolated and knowledge on metabolic pathway activity is still inaccessible. Therefore, the question of whether this organism follows the oxygen-sensitive route, as does the anaerobic bacterium *D. desulfuricans*^32^, also remains unsolved. Using native genes from other organisms, the oxygen-tolerant pathway was integrated and overexpressed as a synthetic route in the model organism *E. coli*^37–39,48^. The authors achieved growth on formate, methanol and CO_2_, thus confirming that this pathway principally supports sufficient flux for cell proliferation. When all other glycine synthesis routes were blocked, and the reductive glycine pathway from formate to glycine in *S. cerevisiae* was overexpressed, the resultant strain produced sufficient glycine from formate and CO_2_ to grow on glucose^34^. This indicates that the flux to glycine for growth solely on formate and CO_2_ without glucose could not be achieved even with a strongly overexpressed pathway. This concurs with our findings that the glycine cleavage (Gcv) system is reversible and that native Gcv and Mis enzymes are active in yeast. The metabolic activity of pyruvate carboxylase in *K. phaffii* is obvious and was metabolically verified by reverse labelling of fully ^13^C-labelled biomass with ^nat^C CO_2_^15^. Our DasKO strain confirmed these findings.

Although *K. phaffii* has the metabolic and genomic capability to assimilate and grow on methanol via the formate-fixing reductive glycine pathway, the organism has evolved to use the formaldehyde-fixing xylulose 5-phophate pathway as the main route for methanol utilisation. Where growth was achieved via the Gcv system, all experiments described in the literature^34,37,39^ show that elevated CO_2_ concentrations are a prerequisite and we could confirm that increased CO_2_ levels boost the pathway activity. *K. phaffii* was isolated from trees^5^, so it has obviously evolved under atmospheric CO_2_ concentrations that appear to be too low to achieve sufficient glycine synthesis via the Gcv system for C1-mixotrophic growth.

Methanol and formate are considered valuable substrates for sustainable biotechnology-based production of chemicals and also for food and feed proteins. Both methanol and formate can be produced electrochemically from CO_2_^2,3^. The co-assimilation of CO_2_ with methanol or formate would even enable direct carbon capture and reduce greenhouse gas emissions. The discovery of a native functional methanol, formate and CO_2_ assimilation pathway in *K. phaffii* bears huge potential for the design of a chassis cell that can convert C_1_ substrates to pyruvate and oxaloacetate. From these two central metabolites, all carbon backbones of metabolites and microbial biomass are generated. Co-assimilation of CO_2_ with a reduced substrate such as methanol is of special interest for the production of oxidised molecules (such as organic acids) because the reducing equivalents for CO_2_ assimilation in the synthesis pathway are thus directly provided^49,50^. The industrial relevance of the oxygen-tolerant pathway in *K. phaffii* can be pathbreaking, as it can lead to improved product yields on methanol compared to the wildtype while enabling net CO_2_ fixation. Due to better energy efficiency it bears the potential to outperform the recently described CBB cycle strain^15^.Enabling methanol oxidation to formaldehyde by *ADH2*^17^, theoretically up to 2/3 moles of methanol and 1/3 mole of CO_2_ are fixed into 1 mole carbon at the degree of reduction of pyruvate (calculations see Supplementary Note 3).

So far, growth on single-carbon substrates alone via the oxygen-tolerant reductive glycine pathway could only be achieved in model organisms when the Mis enzymes of bacteria were used, which support growth via the tetrahydrofolate pathway, e.g. the serine cycle or the reductive acetyl-CoA pathway^26,27,37,39^. Recently an engineered *E. coli* strain could be evolved to grow with a doubling time of 6.3 hours on formate and 10% CO_2_^51^. Here we demonstrate that the yeast *K. phaffii* can grow on mixed C1 substrates of CO_2_ with either methanol or formate with its own enzyme machinery and without overexpression after rebalancing the utilisation of methylene-THF more towards glycine formation, by deletion of the mitochondrial serine hydroxymethyl transferase gene. The growth rates are still very low, with a doubling time of 14 days at best during the active growth phase. Further metabolic engineering and adaptive evolution hold promise, however, to improve growth markedly in future studies.

## Methods

### Plasmid construction

All plasmids were constructed by Golden Gate cloning^13^. For all knockouts, single-guide RNA plasmids with the Cas9 protein and linear homologous DNA sequences were constructed for complete gene deletions as follows^14^. For the *MIS1-2 & 3* knockout only, a split marker cassette was constructed^10^ (see also Supplementary Method 1). For the gene deletions of *DAS1* and *DAS2*, the guide RNA plasmids and the homology regions, with a 22 bp linker region in between were taken from Gassler et al. ^15^. For the knockouts of *GCV1, GCV2, SHM1, SHM2, MIS1-1*, and *MIS1-2 & 3*, the homology regions were amplified by PCR (NEB, Q5 high-fidelity DNA polymerase) from wildtype genomic DNA of CBS7435 used previously^15^. The ligated homologous regions, the natMX-splitmarker for *MIS1-2 & 3* and the overexpression cassette for CRISPR integration were also amplified for transformation with PCR. The single guide RNA recognition site (sequences Supplementary Table 1) was generated by overlap extension PCR and cloned into CRISPi plasmids^14^. Promoters, terminators and plasmid backbones used for overexpression were constructed in previous studies^13^. The coding sequences of *GCV1, GCV2, GCV3, LPD1, MIS1-1, SHM1, SHM2, and CHA1* were amplified from the genomic DNA mentioned above. *ADE3* was amplified from genomic DNA of *S. cerevisiae* (S288C). For *Fhs, FchA and MtdA* the native *M. extorquens* sequence was codon optimized (see Supplementary Data 1) and ordered from Twist Bioscience HQ, USA. For the final overexpression plasmid constructs see Supplementary Table 2.

### Strain construction

All knockouts in this study were constructed by CRISPR/Cas9-based homology-directed recombination^14^. 500 ng guide RNA plasmid and 3-5 µg linear homologous DNA sequence were transformed in *K. phaffii* (*P. pastoris*) CBS7435^47^ by electroporation^10^. The DasKO strain was constructed within a single transformation with both guide RNA plasmids and linear homologous DNA sequences. This strain served as the foundation for all further strain constructions in this study, and additional knockouts were performed consecutively. The *mis1-2 & 3* knockout was conducted by combining a splitmarker-method^10^ with CRISPR/Cas9 and supplementing YPD plates with 10 mmol L^-1^ hypoxanthine (further details in Supplementary Method 1). Screening for the correct gene deletion without gene reintegration was performed by colony PCRs using primer pairs that bound outside the homology regions and inside the gene (primer listed in Supplementary Table 3). For the creation of overexpression strains, ~3 µg of the final overexpression plasmids were linearised with either *SmaI* or *AscI* (New England Biolabs) and transformed consecutively into the DasKO strain as mentioned above. Integration in the correct locus was verified with the primer pair listed in Supplementary Table 3.

### Labelling experiments & metabolic sampling

10 mL YPD (10 g L^-1^ yeast extract, 20 g L^-1^ soy peptone, 22 g L^-1^ D-glucose) precultures were inoculated with a single colony and shaken at 25 °C and 180 rpm overnight. A following 100 mL Yeast nitrogen base without amino acids (YNB) (Sigma-Aldrich GmbH, with 10 g L^-1^ (NH_4_)_2_SO_4_, 0.1 mol L^-1^ potassium-phosphate buffer, pH = 6) batch culture was inoculated to an OD_600_ of 1 (25 °C, 180 rpm overnight). For methanol and formate forward labelling, 18 g L^-1 nat^C-glycerol (Carl Roth GmbH) and for reverse labelling, 0.79% (v/v) fully labelled ^13^C-glycerol (99atom% ^13^C, CortecNet) were used as carbon sources in the batch cultures. For ShmKO, the batch culture was also conducted on YPD and extended to 48 hours to gain sufficient biomass. The MisKO pre and batch cultures were conducted in YPD with 5 mmol L^-1^ hypoxanthine (Sigma-Aldrich GmbH) and 100 mg L^-1^ nourseothricin. Methanol and CO_2_ reverse labelling cultivations were performed in YNB medium with 1% (v/v) ^13^C-methanol (99atom% ^13^C, CortecNet), formate labelling in YNB medium with 30 mmol L^-1 13^C-sodium formate (99atom% ^13^C, Sigma-Aldrich GmbH). For the labelling cultivations with ^nat^C-glycine, glycine (Merck KGaA) was added at a concentration of 20 mmol L^-1^ in addition to methanol. The biomass of the batch cultures was washed and inoculated at an OD_600_ of 50 (which corresponds to 10 g L^-1 nat^C-cell dry weight) for methanol and CO_2_ reverse labelling, or at an OD_600_ of 25 (which corresponds to 5 g L^-1^ cell dry weight) for formate labelling. Shm1KO and the corresponding DasKO-control labelling was performed for 24 hours at an OD_600_ of 25 with the addition of 5% naturally distributed CO_2_. For experiments with metabolite sampling points at 2 h, 24 h and 72 h, the starting volume was set to 16 mL. ^13^C-methanol was adjusted to 1% (v/v) after 24 h, 48 h and 58 h. ^13^C-formate concentration was adjusted to 30 mmol L^-1^ after 7 h, 17 h, 22 h, 30 h, 41 h and 52 h (for methanol and formate feeding profiles see Supplementary Fig. 8, at each sample point HPLC measurements^50^ were conducted to assess the carbon source concentration). Each metabolic sample had 3 mL, additional samples for methanol measurements 1 mL and for formate measurements 300 µL. Experiments with metabolic sampling at 24 h only had an initial volume of 10 mL. Methanol and formate labelling was conducted at 25 °C at atmospheric gas conditions, CO_2_ reverse labelling at 30 °C in 5% ^nat^C-CO_2_ shaken at 180 rpm. All forward labelling experiments were conducted in biological duplicates with parallel ^nat^C-carbon source control experiments.

Metabolic sampling was performed with cold methanol quenching^17,52^. Briefly, 1 or 2 mL culture (corresponding to 10 mg ^nat^C-cell dry weight) was quenched in the 4-fold volume of quenching solution (60% methanol (Sigma-Aldrich GmbH), 125 mmol L^-1^ TRIS-HCl (Carl Roth GmbH), 55 mmol L^-1^ NaCl (Merck KGaA, pH 8.2; T = −27 °C). The mixture was immediately vortexed for 4 s and rapidly filtered through cellulose acetate filters (0.45 µm, Sartorius Stedim Biotech GmbH). The biomass was washed on the filter with 10 mL 60% methanol and stored at -70 °C until metabolite extraction.The labelling experiment of the Mut^-^ strain including sampling and extraction was performed within the study of Zavec et al. ^17^.

### Sample preparation & GC-TOFMS analysis of intracellular metabolites

For gas chromatography time-of-flight mass spectrometry (GC-TOFMS) isotopologue distribution analysis, the quenched cells were extracted using boiling ethanol extraction as established in our laboratory for *K. phaffii*^16,53^. Briefly, 4 mL 75% ethanol (HPLC-grade, Sigma-Aldrich GmbH) at 85 °C was added to the cell pellet on the filter. Samples were vortexed, heated for 3 min at 85 °C and rapidly cooled on dry ice before centrifugation at 4000 × *g* and -20 °C for 10 min. The supernatants were dried in a vacuum centrifuge, and dry extracts were stored until analysis at -80 °C. Before analysis, the extracts were reconstituted in 1 mL MS-grade water for 30 min at room temperature.

All metabolites were derivatised using a sample preparation robot (MPS2, Gerstel) for automated just-in-time online derivatisation prior to gas chromatography time-of-flight mass spectrometry (GC-QTOFMS, 7200B, Agilent Technologies).

For some metabolites (primarily phosphorylated ones, see Supplementary Data 2), a method based on ethoximation and trimethylsilylation followed by gas chromatography chemical ionisation time-of-flight mass spectrometry (GC-CITOFMS) was used^43^. This method was originally designed and developed for the *K. phaffii* wildtype on glucose by Mairinger et al. ^43^. Minor adaptions at the GC-MS data acquisition level were made and significant changes were necessary for subsequent data analysis. As the cultivation of the XuMP knockout strain (DasKO) on methanol and the use of formate as the carbon source led to profound changes in analyte concentrations and matrix composition of the labelling samples, the chromatographic and mass spectrometry-related background differed significantly from previously analysed *K. phaffii* samples. Consequently, the method had to be further developed and optimised. Technical changes included: (i) slowing down the GC-temperature program to reduce interferences (70 °C hold for 1 min, 15 °C min^-1^ to 190 °C, 5 °C min^-1^ to 225 °C, 3 °C min^-1^ to 260 °C, 20 °C min^-1^ to 310 °C, hold for 3 min); and (ii) the use of an Agilent split/splitless injector with a splitless gooseneck liner with glass wool (Agilent). 350 µL of the reconstituted extract was dried in 400 µL inserts in 1.5 mL chromatography vials after the addition of 40 µL ethoxyamine hydrochloride in pyridine (*c* = 20 g L^-1^). Vials were crimped with magnetic caps before placing the samples in the autosampler at 7 °C.

To cover all metabolites of importance and to extend the biological information through positional information obtained via specific fragmentation in the electron ionisation (EI) ion source, an additional GC-MS-based method was implemented, further developed and optimised with respect to measurement and data evaluation (Supplementary Method 2). For amino acid and organic acid analysis, TBDMS- (*tert*-butyldimethylsilyl-) derivatisation followed by GC-EITOFMS in split mode (split 1:50, Agilent straight split liners with glass wool) was used^15^. For the analysis of metabolites of low concentration (e.g., glycerate), some derivatised samples were reinjected in splitless mode after changing to an Agilent splitless gooseneck liner with glass wool (see Supplementary Table 4). This method was used in the study of Gassler et al. ^15^ for amino acid analysis only, was extended to cover more metabolites and was applied with minor technical adaptions. More specifically, the final hold time of the temperature program was extended from 4 to 9 minutes to enable the elution of citrate. 150 µL of the reconstituted extract was dried in inserts for this procedure.

### GC-TOF-MS data evaluation

For isotopologue distribution analysis, the exact masses of a number of adducts and fragments of all target analytes were calculated and used for data analysis in both profile and centroid mode (Supplementary Data 3 & 4). Extracted ion chromatograms (EICs) were integrated with Agilent Technologies MassHunter Workstation Quantitative Analysis for TOF (Build 10.1.733.0). The extracted ion chromatograms (EICs) of all *m/z* ratios, the integration of the peaks and the corresponding mass spectra were visually inspected and rejected if mass spectral or chromatographic interferences were detected, or if peaks were saturated. For cases where the automated integration was not satisfactory, peaks were reintegrated manually. The resultant peak areas were corrected for natural heavy isotopes of H, N, O, Si, S atoms of the derivatised molecule and C isotopes originating from derivatisation (nonmetabolite carbon atoms) using the ICT correction toolbox v.0.04^54^. Carbon isotopologue fractions were calculated (with *n* = number of carbon atoms in the metabolite, *A*_*i*_ = ICT corrected peak area of isotopologue i, *i.e*., an isotopologue containing i numbers of ^13^C atoms) according to Eq. 1:

1 $${{Carbon} \, {isotopologue}{fraction}}_{i}=\frac{{A}_{i}}{{\sum }_{i=0}^{n}{A}_{i}}$$

Several fragments and adducts were evaluated in profile and centroid mode for each metabolite, and the corresponding ^13^C carbon isotopologue fractions were calculated. The decision on which fragments or adducts to finally evaluate for different time points and strains was based on the trueness of the results. For this purpose, the isotopologue fractions obtained for ^nat^C labelled metabolites measured in ^nat^C extracts (average of 2 biological replicates) were compared with the calculated natural isotopologue fractions (calculated via https://www.envipat.eawag.ch/^55^). This was achieved in two ways: (i) the uncorrected measured ^nat^C isotopologue distributions (not ICT corrected, hence corresponding to the isotopologue fraction of the entire derivatised molecule with *A*_*i*_ corresponding to the uncorrected peak area of respective isotopologues) were compared with the isotopologue fractions calculated via Envipat for the entire derivatised compound; and (ii) the carbon isotopologue fractions as obtained after ICT correction were compared to the isotopologue fractions calculated via Envipat for the respective number of carbon atoms in the metabolite. Fragments or adducts were only considered for further evaluation if both the deviation between measured and calculated isotopologue fraction and the deviation between measured and calculated carbon isotopologue fraction was less than 5%. For each strain, time point, and metabolite, the fragment or adduct in centroid or profile mode that was selected for further data interpretation had the lowest deviations of the (carbon) isotopologue fractions from the calculated theoretical values and the lowest standard deviation for ^nat^C and ^13^C replicates (chosen adducts/fragments and type of data (profile/centroid) are summarised in Supplementary Data 2). ^13^C carbon isotopologue fraction data in Fig. 2 and Fig. 3 are shown as the average of biological duplicates with error bars representing the standard deviation calculated from duplicates multiplied by the correction factor of 1.253314, as proposed by Roesslein et al. ^56^. ^nat^C carbon isotopologue fraction data are presented as mean and standard deviation of n replicates of the selected fragments or adducts from naturally distributed samples.

### Genome mining for alternative methanol assimilation genes

Genes and enzymes for the reactions of interest were either located in the database KEGG (https://www.genome.jp/kegg/pathway.html#metabolism) or alternatively, enzymes were searched via the corresponding metabolites in Expasy´s chemical compound search (https://enzyme.expasy.org/enzyme-bycompound.html); related genes were determined via the Pichia genome database^47^ (www.pichiagenome.org). Non-annotated enzymes were further assessed for presence in *S. cerevisiae* (https://www.yeastgenome.org/). The homologous gene was either directly located in the Pichia genome database or identified by NCBI´s BLAST, as shown in Supplementary Fig. 9-11) (https://blast.ncbi.nlm.nih.gov/Blast.cgi?PAGE_TYPE=BlastSearch).

### Growth experiments

Cultivations were conducted in 100 mL shake flasks with working volumes of between 8 and 25 mL, containing YNB medium. Precultures and batch cultures of the knockout strains were conducted as in the labelling experiments above. For the overexpression strains the batch culture was omitted. The carbon sources of the test culture were 18 g L^-1^ glycerol, 20 mmol L^-1^ glycine, 20 mmol L^-1^ serine (Carl Roth GmbH), 30 mmol L^-1^ sodium formate (Carl Roth GmbH) or 4 g L^-1^ methanol (Carl Roth GmbH) for the first 24 h. The concentration was then maintained at 8 g L^-1^ methanol or 30 mmol L^-1^ sodium formate by feeding every 2-3 days. Cultures were initiated with an optical density at 600 nm (OD_600_) between 0.5 and 1. Flasks were incubated at 25 °C under atmospheric conditions, at 30 °C and 25 °C with 5% CO_2,_ and at 25 °C with 10% CO_2_ whilst shaking at 180 rpm. The evaporated culture volume between sample points was compensated by the addition of water. OD_600_ and methanol or formate concentrations (HPLC measurements^17^) were monitored every 2-3 days. The ShmKO test cultures on 18 g L^-1^ glycerol, with optional 20 mM glycine, 8 g L^-1^ methanol or 30 mM sodium formate and 5% CO_2_ were performed at 30 °C. Batch cultures were omitted and precultures were performed in YPG (10 g L^-1^ yeast extract, 20 g L^-1^ soy peptone, 18 g L^-1^ glycerol) at 30 °C. The experiments were conducted in duplicates if not stated differently in the figure legends. 100 mg L^-1^ nourseothricin, 200 mg L^-1^ hygromycin, 500 mg L^-1^ geneticin and 25 mg L^-1^ or 50 mg L^-1^ zeocin were added according to the corresponding resistance markers of the overexpression strains to the precultures.

### Reporting summary

Further information on research design is available in the Nature Portfolio Reporting Summary linked to this article.

### Supplementary information

Supplementary Information

Description of Additional Supplementary Files

Supplementray Data 1

Supplementary Data 2

Supplementary Data 3

Supplementary Data 4

Reporting Summary

### Source data

Source Data

## Data Availability

Data supporting the findings of this work are available within the paper and its Supplementary Information files. A reporting summary for this Article is available as a Supplementary Information file. Source data are available at figshare [10.6084/m9.figshare.24224881]^57^ and provided with this paper. Source data are provided with this paper.
