# Supplementary material for: The oxygen-tolerant reductive glycine pathway assimilates methanol, formate and CO2 in the yeast Komagataella phaffii
Source: Nat Commun. 2023 Nov 27;14:7754. doi: 10.1038/s41467-023-43610-7 (PMC10682033; doi:10.1038/s41467-023-43610-7)
Supplement: Supplementary file 1 — Supplementary Information [file 41467_2023_43610_MOESM1_ESM.pdf]

# The oxygen-tolerant reductive glycine pathway assimilates methanol, formate and $CO_2$ in the yeast *Komagataella phaffii*

Mitic *et al.*

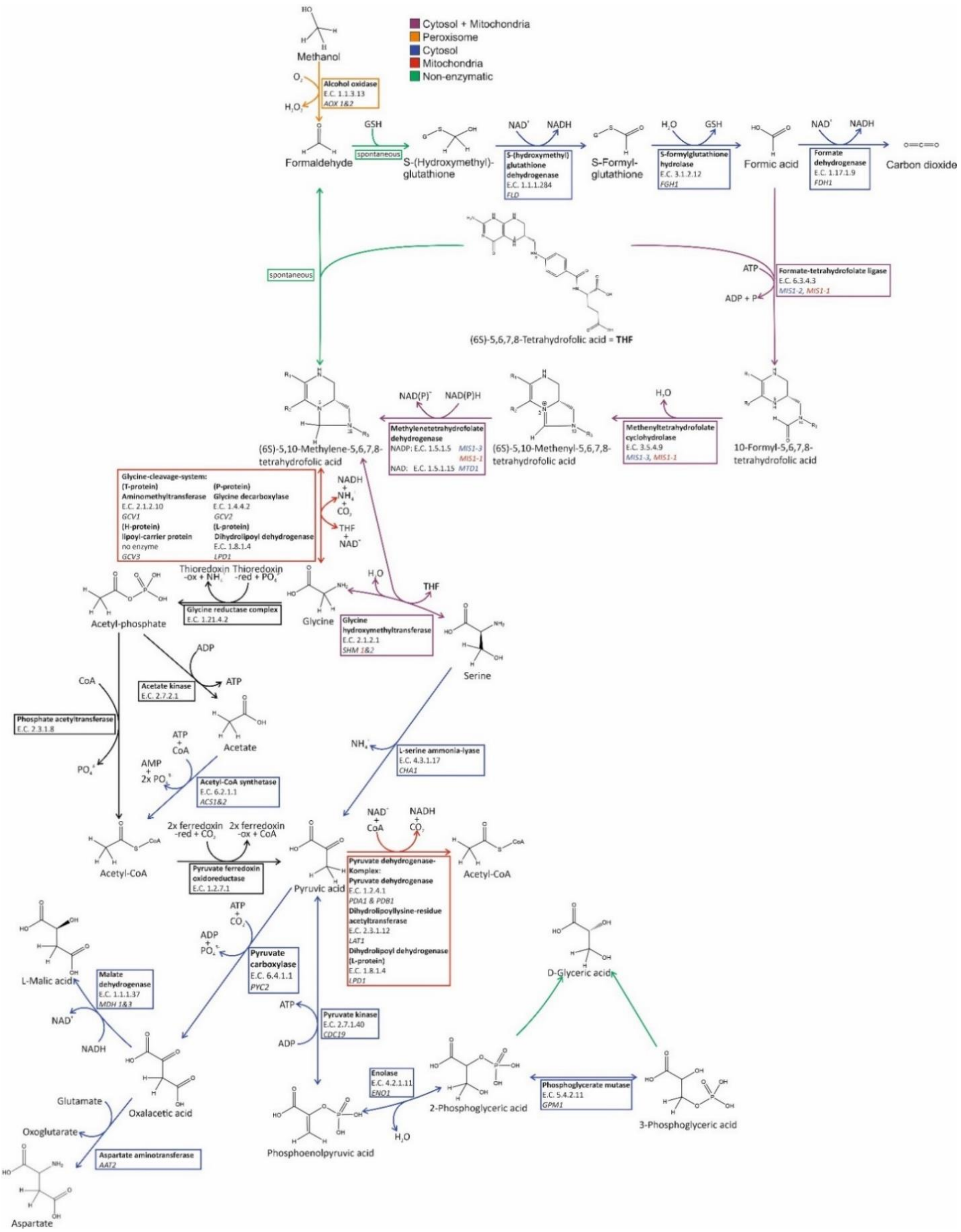


**Supplementary Fig. 1. Detailed reductive glycine pathway.** Metabolite structures, enzyme annotations, gene annotations and compartment localization in *K. phaffii*; $O_2$-sensitive through acetyl-P, $O_2$- tolerant pathway over serine.

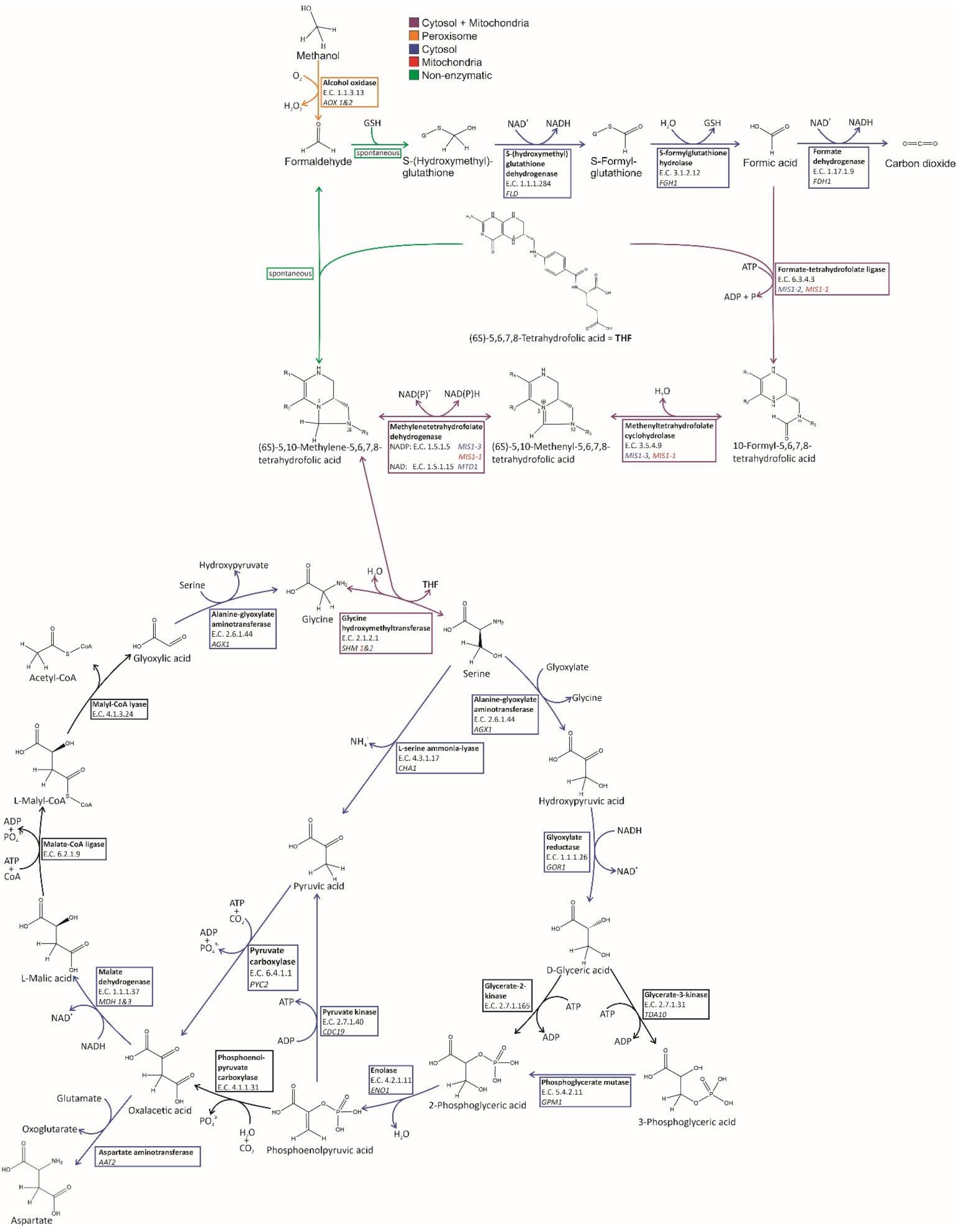


**Supplementary Fig. 2. Detailed pathways of the serine cycle.** Metabolite structures, enzyme annotations, gene annotations and compartment localization in *K. phaffii*; the native pathway is shown as a cycle, alternative routes and shortcuts are shown as straight reactions.

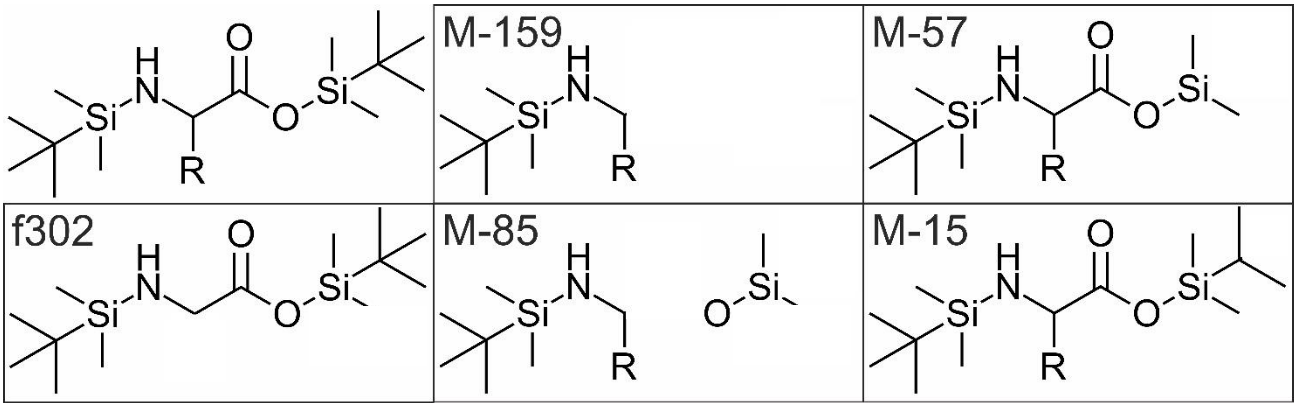


**Supplementary Fig. 3. Amino acid fragmentation patterns.** GC-EI-TOF-MS with TBDMS derivatisation: DC (decarboxylated) corresponds to a mass loss of 159 or 85 Da, BB (amino acid backbone) corresponds to the fragment 302. The fragments M-57 and M-15 represent the full amino acid. See Supplementary Data 2&3. [1,2]

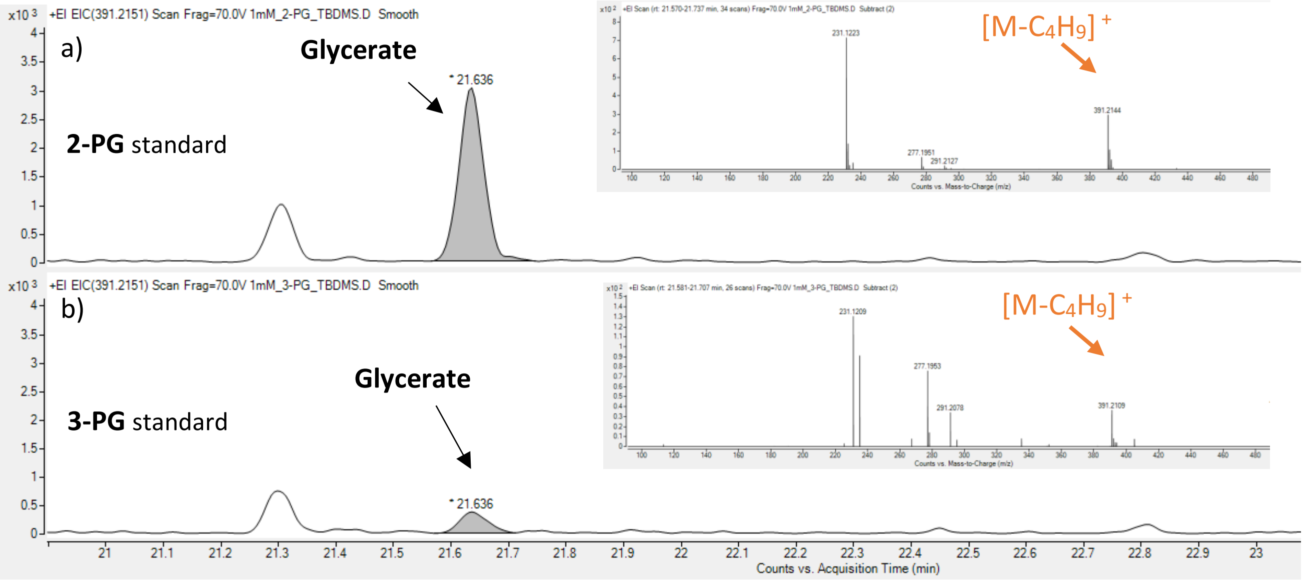


**Supplementary Fig. 4. 2-PG and 3-PG degradation to glycerate shown by TBDMS GC-EI-TOFMS (split injection 1:50).** chromatogram and mass spectrum of the glycerate peak a) in a 2-PG standard ($c$ = 1 mmol $L^{-1}$) and b) in a 3-PG standard ($c$ = 1 mmol $L^{-1}$).

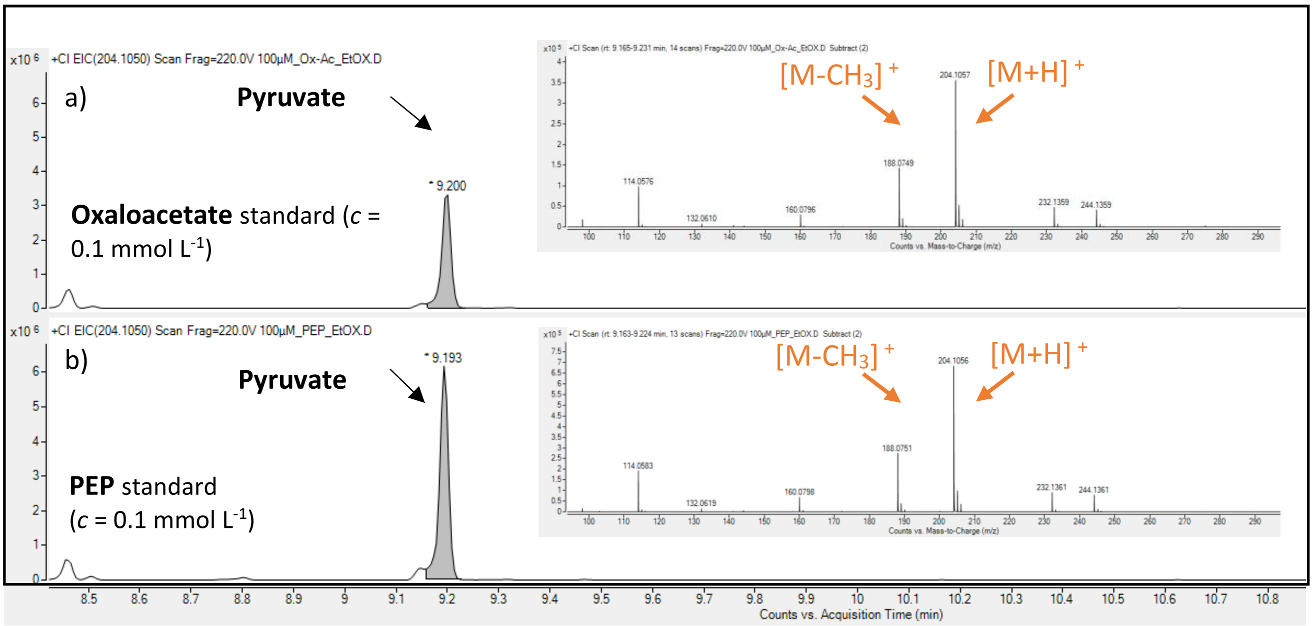


**Supplementary Fig. 5. Oxaloacetate and PEP degradation to pyruvate shown by EtOx/TMS GC-CI-TOFMS (splitless injection).** chromatogram and mass spectrum of the pyruvate peak a) in an oxaloacetate standard ($c$ = 0.1 mmol $L^{-1}$) and b) in a phosphoenolpyruvate standard ($c$ = 0.1 mmol $L^{-1}$)

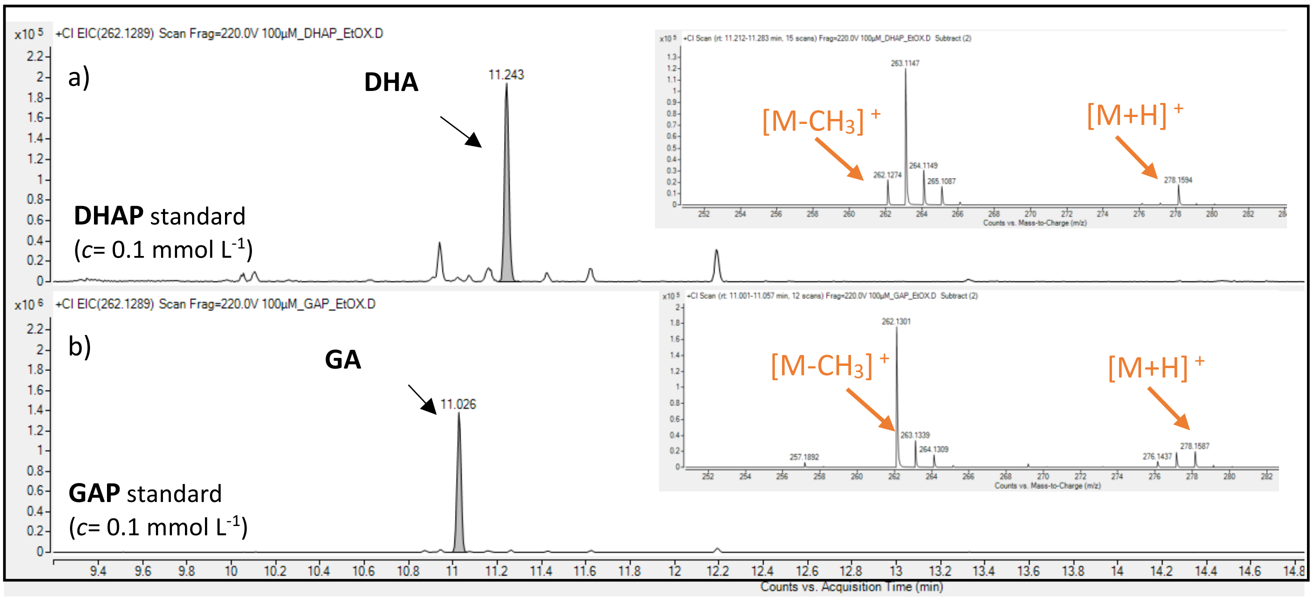


**Supplementary Fig. 6. GAP & DHAP degradation to GA & DHA shown by EtOx/TMS GC-CI-TOFMS (splitless injection).** chromatogram and mass spectrum a) of the dihydroxyacetone peak in a dihydroxyacetone-phosphate standard ($c$ = 0.1 mmol $L^{-1}$) and b) of the glyceraldehyde peak in a glyceraldehyde phosphate standard ($c$ = 0.1 mmol $L^{-1}$).

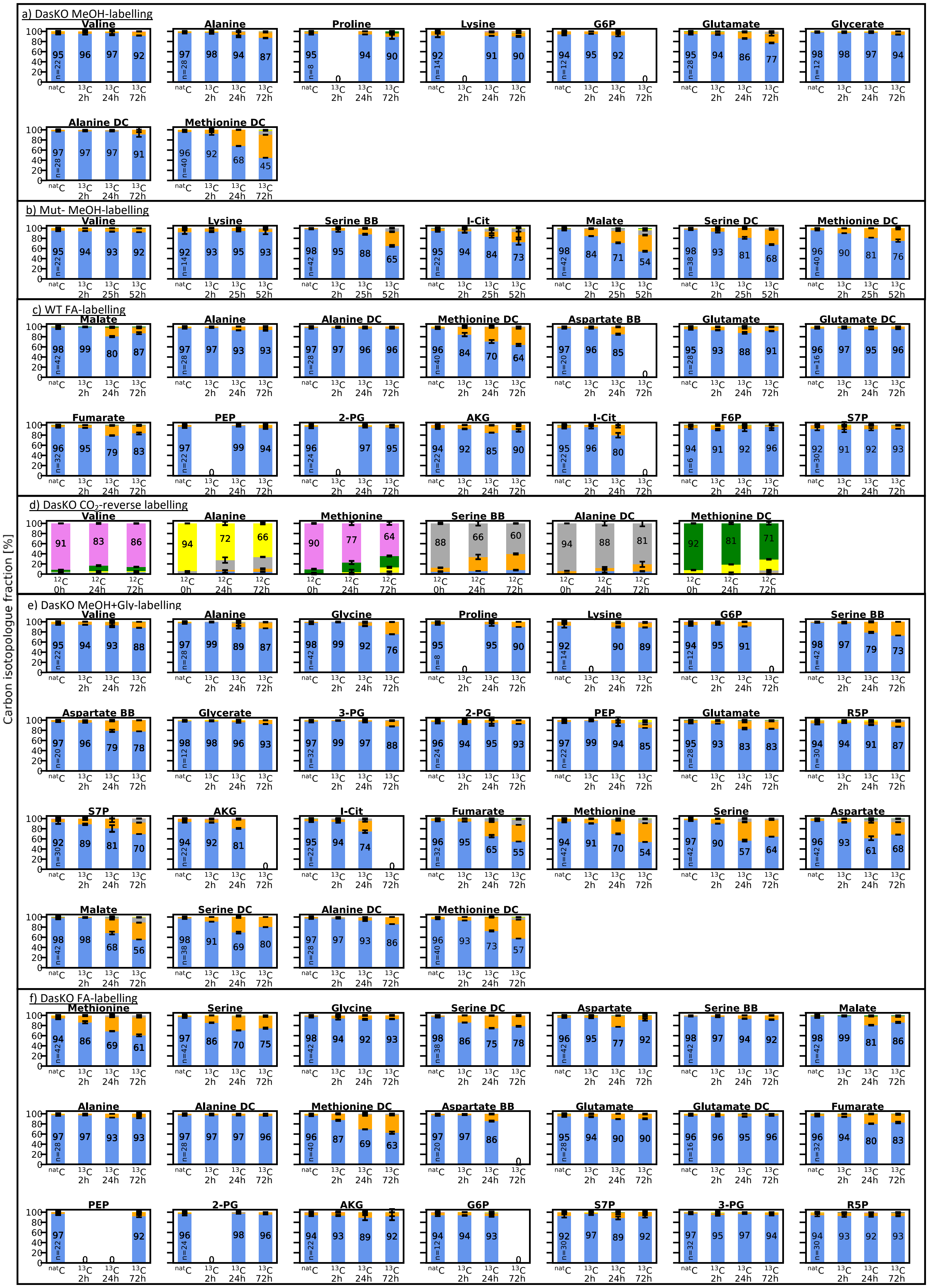


**Supplementary Fig. 7. Carbon isotopologue distribution analysis.** For strain genotypes see Table 1. n=2 for labelling data if not specified differently. Error bars represent corrected standard deviations of mean values.

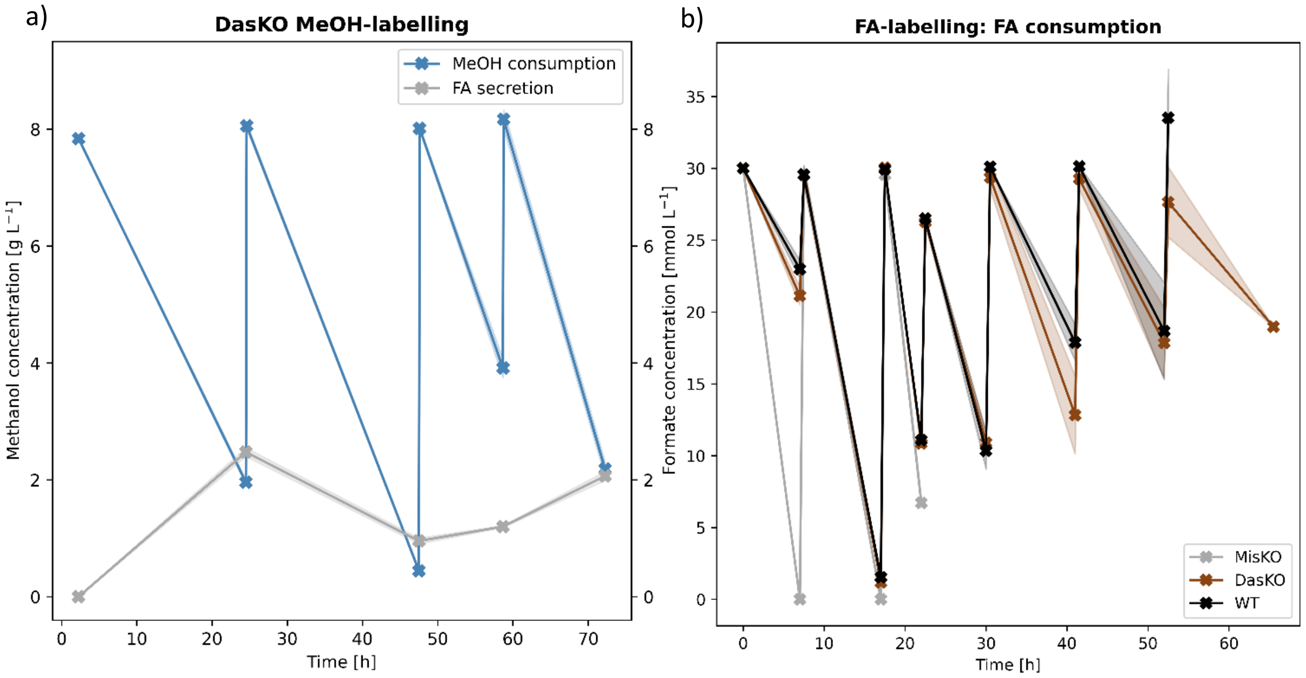


**Supplementary Fig. 8. Carbon source consumption and secretion profile for labelling experiments.** Methanol and formate concentration timelines for a) methanol consumption during DasKO methanol-labelling and corresponding formate secretion/reconsumption in the supernatant, b) formate consumption during formate labelling.

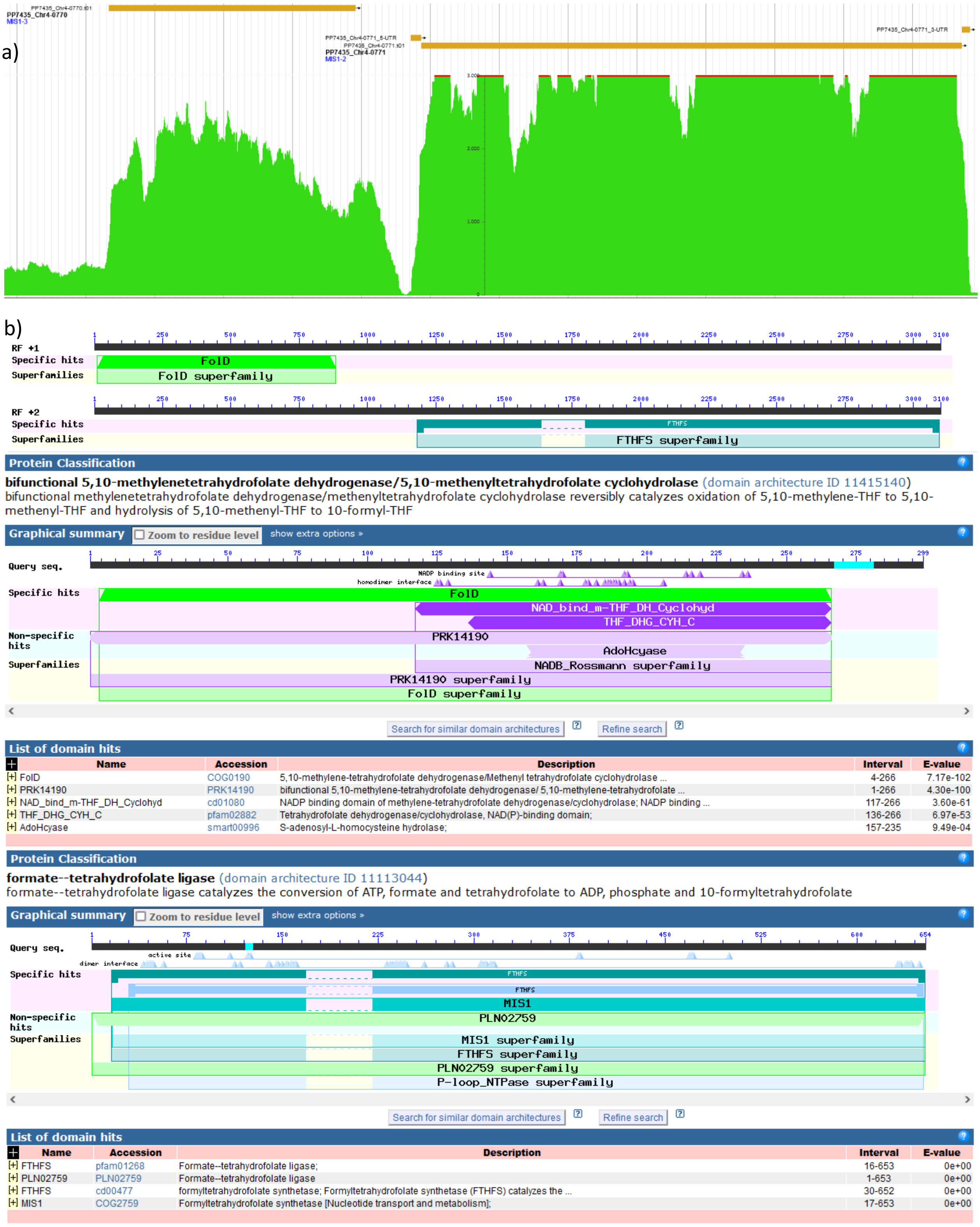


| Name | Accession | Description | Interval | E-value |
|---|---|---|---|---|
| [+] FolD | COG0190 | 5,10-methylene-tetrahydrofolate dehydrogenase/Methenyl tetrahydrofolate cyclohydrolase ... | 4-266 | 7.17e-102 |
| [+] PRK14190 | PRK14190 | bifunctional 5,10-methylene-tetrahydrofolate dehydrogenase/ 5,10-methylene-tetrahydrofolate ... | 1-266 | 4.30e-100 |
| [+] NAD_bind_m-THF_DH_Cyclohyd | cd01080 | NADP binding domain of methylene-tetrahydrofolate dehydrogenase/cyclohydrolase; NADP binding ... | 117-266 | 3.60e-61 |
| [+] THF_DHG_CYH_C | pfam02882 | Tetrahydrofolate dehydrogenase/cyclohydrolase, NAD(P)-binding domain; | 136-266 | 6.97e-53 |
| [+] AdoHcyase | smart00996 | S-adenosyl-L-homocysteine hydrolase; | 157-235 | 9.49e-04 |

| Name | Accession | Description | Interval | E-value |
|---|---|---|---|---|
| [+] FTHFS | pfam01268 | Formate--tetrahydrofolate ligase; | 16-653 | 0e+00 |
| [+] PLN02759 | PLN02759 | Formate--tetrahydrofolate ligase | 1-653 | 0e+00 |
| [+] FTHFS | cd00477 | formyltetrahydrofolate synthetase; Formyltetrahydrofolate synthetase (FTHFS) catalyzes the ... | 30-652 | 0e+00 |
| [+] MIS1 | COG2759 | Formyltetrahydrofolate synthetase [Nucleotide transport and metabolism]; | 17-653 | 0e+00 |

**Supplementary Fig. 9. Cytosolic homologs MIS1-2&3 of *K. phaffii* are separated.** a) mRNA enriched coverage histogram of *MIS1-2&3* of *K.phaffii* (http://pichiagenome-ext.boku.ac.at:8080/apex/f?p=100:23:1507211694349::NO). b) protein alignment with NCBI´s BLASTp of *MIS1-2* and *MIS1-3* (https://www.ncbi.nlm.nih.gov/Structure/cdd/wrpsb.cgi?RID=B8U6Y2FS013&mode=all, https://www.ncbi.nlm.nih.gov/Structure/cdd/wrpsb.cgi?RID=B8U7N3P2013&mode=all).

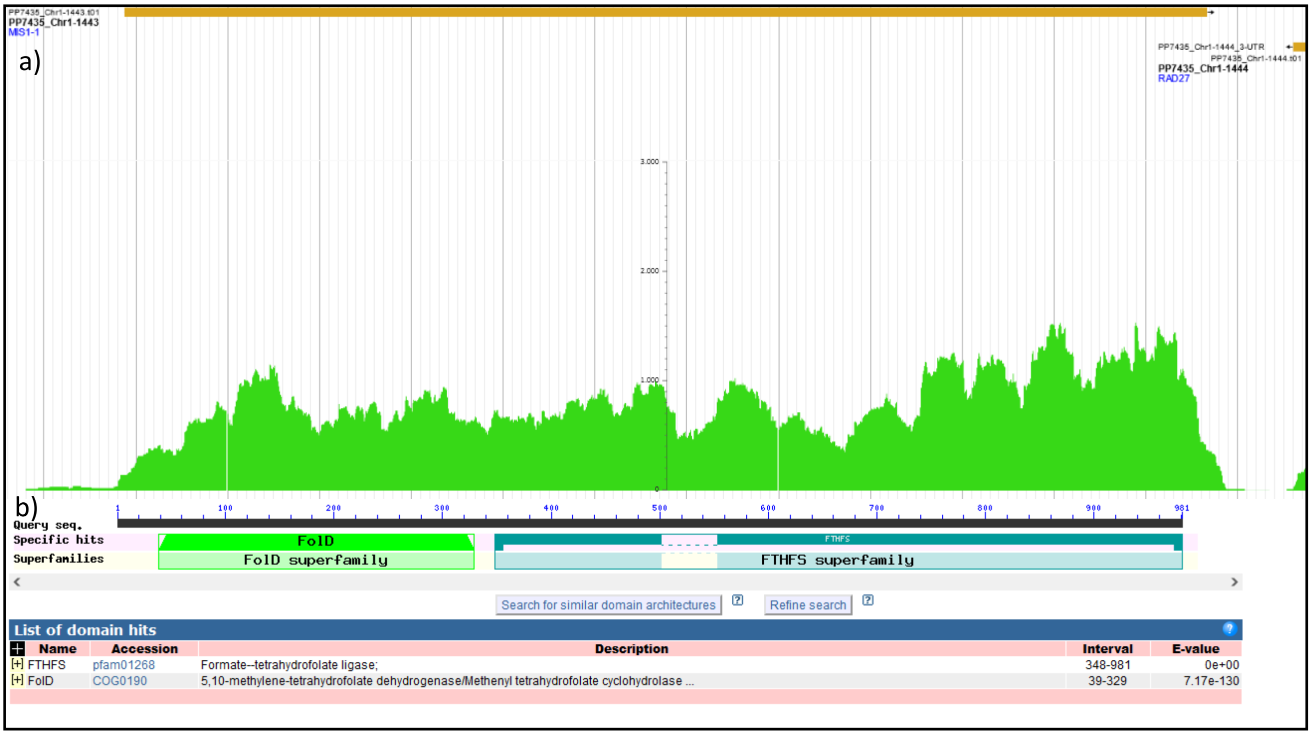


**Supplementary Fig. 10. Mitochondrial homolog *MIS1-1* of *K. phaffii* has no separation.** a) mRNA enriched coverage histogram of *MIS1-1* of *K. phaffii* (http://pichiagenome-ext.boku.ac.at:8080/apex/f?p=100:23:1507211694349::NO). b) protein alignment with NCBI´s BLASTp of MIS1-1 (https://www.ncbi.nlm.nih.gov/Structure/cdd/wrpsb.cgi?RID=B8WME1V6016&mode=all).

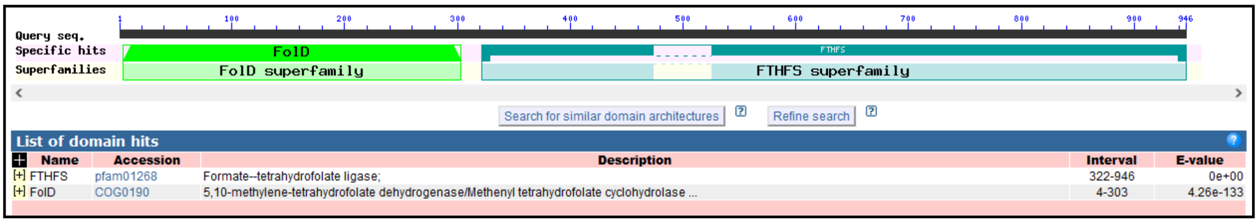


**Supplementary Fig. 11. Cytosolic homolog *ADE3* of *S. cerevisiae* has no separation.** Protein alignment with NCBI´s BLASTp of *ADE3* (https://www.ncbi.nlm.nih.gov/Structure/cdd/wrpsb.cgi?RID=B8WZ38XT013&mode=all).

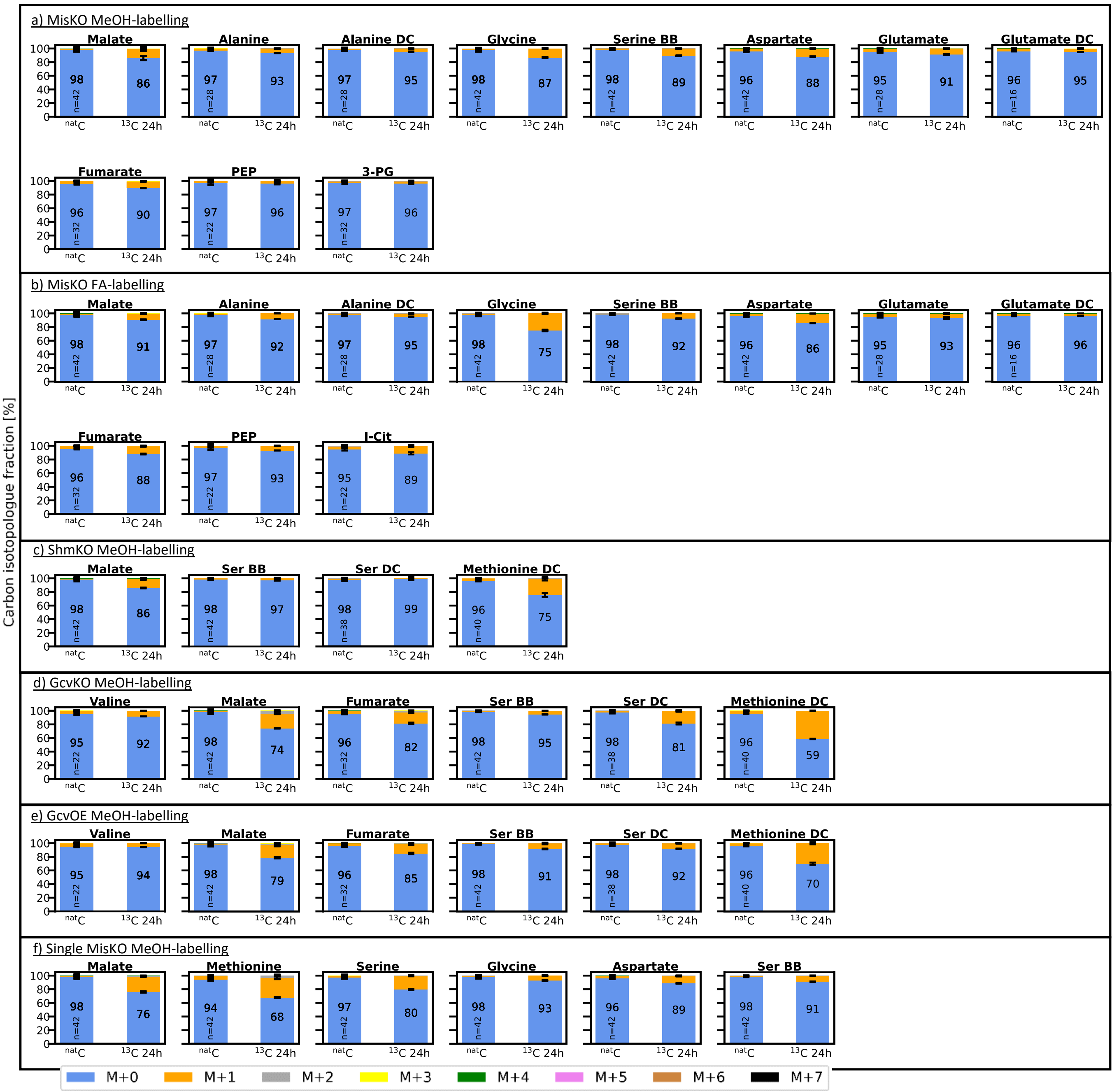


**Supplementary Fig. 12. Additional results of carbon isotopologue distribution analysis of further knockout strains.** For strain genotypes see Table 1. n=2 for labelling data if not specified differently. Error bars represent corrected standard deviations of mean values.

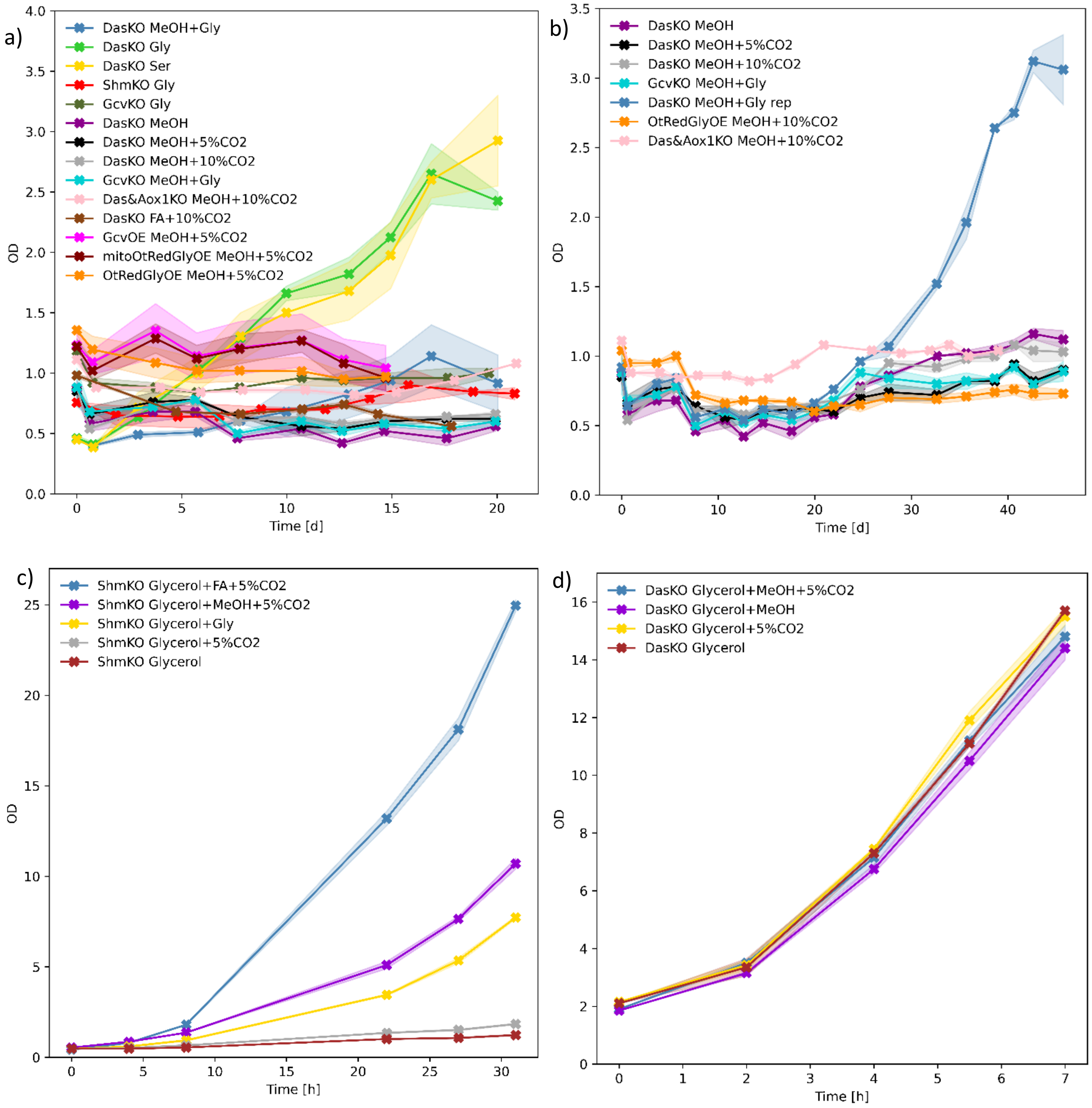


**Supplementary Fig. 13. Growth experiment results.** a) non-normalized $OD_{600}$ of main manuscript Fig.4a; b) long cultivations and repeatability study; c) non-normalized $OD_{600}$ of main manuscript Fig.4b; d) comparison of experiment to (c) with an Shm active strain within the same amount of cell-doublings.

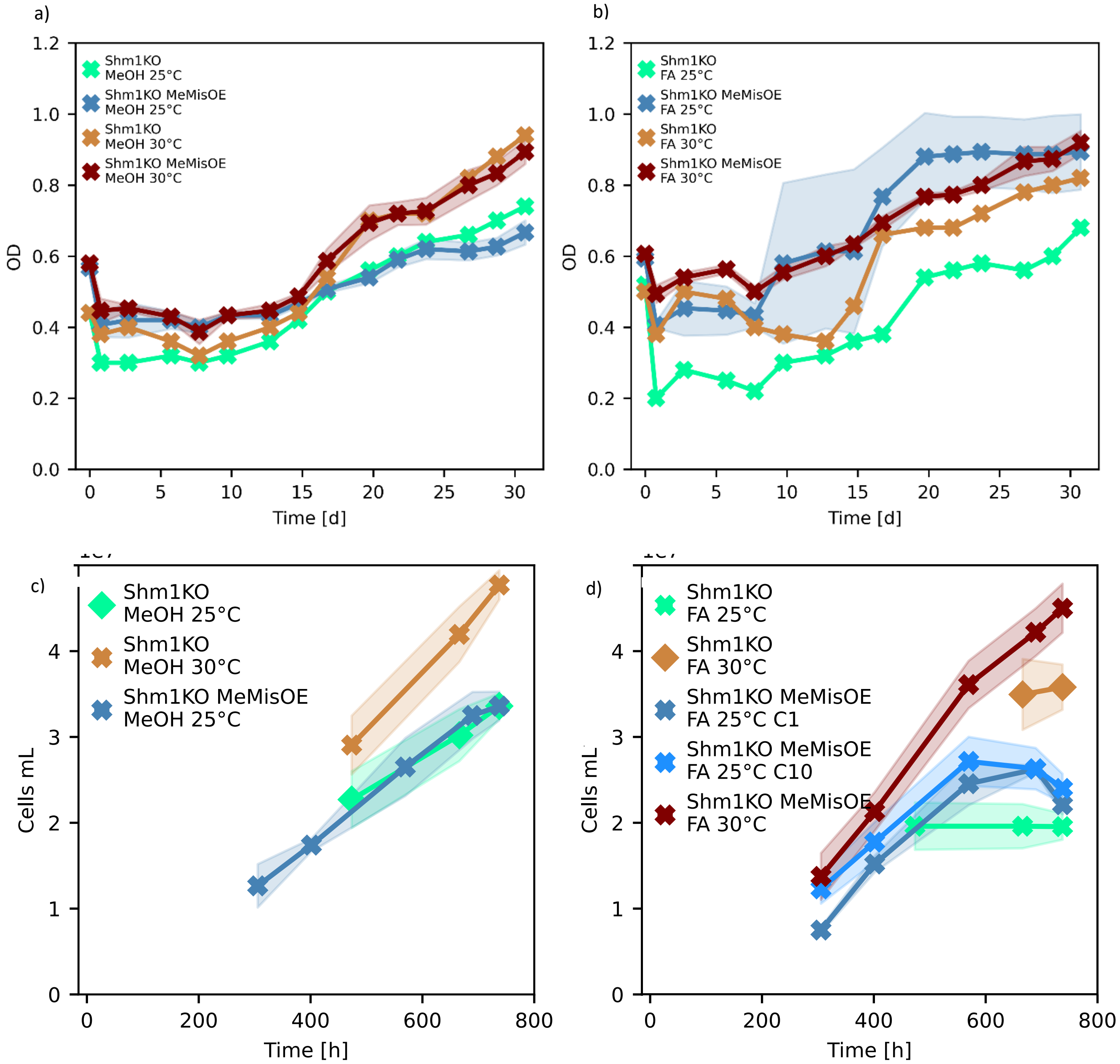


**Supplementary Fig. 14. Growth experiment results of *SHM1* knockout strain.** a) non-normalized $OD_{600}$ of main manuscript Fig.4c and b) non-normalized $OD_{600}$ of main manuscript Fig. 4d. c & d: Cell counts of cultivations of the Shm1KO and the Shm1KO MeMisOE strains using c) methanol and d) formate as carbon source. Cell counting was performed using a Neubauer improved counting chamber. 10 µL were loaded into the chamber and 5 medium squares for each sample were counted and multiplied by $2.5 * 10^5$ to obtain the cell count in cells * $mL^{-1}$.

**Supplementary Table 1. Single guide RNA recognition site sequences.**

| **Name** | **Sequence (5'→3')** |
|---|---|
| *GCV1* | TCTTGTAGAAGATACAGCACGGG |
| *GCV2* | GTTGAGATCACACAGAGTGAAGG |
| *SHM1* | CTTCTACATTTCCTGTTCCGGGG |
| *SHM2* | TCAAGAATGAGATTAGCGCCTGG |
| *MIS1-1* | AGCACCGTTAGAAGGCTCGCTGG |
| *MIS1-2&3* | ATGACAAGGGAGAGATTGAAGGG |

**Supplementary Table 2. Overexpression plasmids.**

| |
|---|
| BB3rN_pFDH1_GCV1m_RPL2Att_pDAS2_GCV2m_RPP1Btt_pAOX_LPD1m_RPS2tt_pDAS1_GCV3m_IDP1tt |
| BB3eH_pDAS1_MIS1-1m_IDP1tt_pAOX1_SHM1_RPS2tt_pDAS2_CHA1_RPP1Btt |
| BB3aK_pDAS1_ADE3_IDP1tt_pAOX1_SHM2_RPS2tt |
| BB3eH_pDAS1_ADE3_IDP1tt _pAOX1_SHM2_RPS2tt _pDAS2_CHA1_RPP1Btt |
| BB3eH_pDAS1_Fhs_RPS2tt_pCS1_FchA_IDP1tt_pTEF2_MtdA_DAS1tt |

**Supplementary Table 3. Primer list.**

| **Name** | **Sequence (5’→3’)** |
|---|---|
| DAS1_Seq_ex_fw | ATTCTGTCGAAAATGGAAGCG |
| DAS1_Seq_ex_rev | CACTTGCATCACTGGCT |
| DAS1_Seq_int_fw | GGTCATCAAAACCTTCCGTT |
| DAS1_Seq_int_rev | AGCCTTGATAGAGTTGACATATTG |
| DAS2_Seq_ex_fw | ATGAAAGGGTTACGGGTGTT |
| DAS2_Seq_ex_rev | TGCTGGCTGGTGTATCTCTC |
| DAS2_Seq_int_fw | CGGTGAATTCGTAAAGGATTTGA |
| DAS2_Seq_int_rev | GCAGTATCGACACAAGATGAC |
| GCV1_Seq_ex_fw | CTTGTATTTTCCTTTCAGGGGG |
| GCV1_Seq_ex_rev | GTTGGGAGATACTCTTAATAGTTTCC |
| GCV1_Seq_int_fw | GAGTAGAAGCTCTTATCAAAACTCC |
| GCV1_Seq_int_rev | GCTACAAAAGGAAGTTTGGC |
| GCV2_Seq_ex_fw | GTCTACAAGATTGGTGGTATCG |
| GCV2_Seq_ex_rev | GAAGGAGTTATGAACCTAAACTGG |
| GCV2_Seq_int_fw | CACAGCAAGTTCATGTCTCC |
| GCV2_Seq_int_rev | GAGATCACACAGAGTGAAGG |
| SHM1_Seq_ex_fw | CATTGTGGAACGGTATTTGC |
| SHM1_Seq_ex_rev | CATTCTAAGCATTGGAAAAGATCG |
| SHM1_Seq_int_fw | GAACAGGAAATGTAGAAGCCC |
| SHM1_Seq_int_rev | CTTAGTCGTAACCTGCAAAGG |
| SHM2_Seq_ex_fw | GTTGATGCTGTTTGTTCAGC |
| SHM2_Seq_ex_rev | CTTATCAAGGTTAACGGTTCACC |
| SHM2_Seq_int_fw | CTAAACAGACAATGGGAATTCTCC |
| SHM2_Seq_int_rev | CCAAATCATCAAAGATGAGGTCG |
| MIS1-1_Seq_ex_fw | GGAGATTTGTTTTCAATGGACC |
| MIS1-1_Seq_ex_rev | GAGATTGGACATGAAAGAAACAGG |
| MIS1-1_Seq_int_fw | CTAGAAATAAAGCAGCTAGCACC |
| MIS1-1_Seq_int_rev | CATTGAGATATCCTGGATGAGTAGG |
| MIS1-2&3_Seq_ex_fw | GTGAGCCTTCAATTACCTCG |
| MIS1-2&3_Seq_ex_rev | GAGATATTGTCAGAATTGTCTTCTGC |
| MIS1-2&3_Seq_int_fw | CGGAACTCCGTTTGTCTATAGCGG |
| MIS1-2&3_Seq_int_rev | CTCCCTTGTCATTGACTTCG |
| BB3rN_Seq_fw | CCACCCCGTAGAAAAGATCAA |
| BB3rN_Seq_rev | CGGCCGTTAAAATACTCA |
| BB3eH_Seq_fw | GAAGCACCGGAAGGAA |
| BB3eH_Seq_rev | ACCTATTCAATGACCAACTCCTGG |
| BB3aK_Seq_fw | GAGGGAGCAGGAGTAGG |
| BB3aK_Seq_rev | AGAAGACCGGTCTTGCTA |
| BB3eH_MeMis_rev | TAGGAAGACttGCTTAAGCTTACGGGAAGTC |

**Supplementary Table 4. Overview of GC-MS methods applied for the analysis of cell extracts of strains grown on differentcarbon sources.**

| **Strain name & carbon source(s)** | **EtOX/TMS GC-CI-TOFMS, splitless** | **TBDMS GC-EI-TOFMS, splitless** | **TBDMS GC-EI-TOFMS, split1:50** |
|---|---|---|---|
| DasKO MeOH - labelling | x | x | x |
| DasKO MeOH+Gly - labelling | x | x | x |
| DasKO $CO_2$ - labelling | x | | x |
| DasKO FA - labelling | x | | x |
| GcvKO MeOH - labelling | | | x |
| ShmKO MeOH - labelling | | | x |
| Single MisKO MeOH - labelling | | | x |
| MisKO MeOH - labelling | x | | x |
| MisKO FA - labelling | x | | x |
| GcvOE MeOH - labelling | | | x |
| Mut- MeOH - labelling | Zavec *et al.* [5] | | x |
| WT FA-labelling | x | | x |
| Shm1KO MeOH – labelling + 5% $^{nat}CO_2$ | | | |
| DasKO MeOH – labelling + 5% $^{nat}CO_2$ | | | x |

## Supplementary Note 1. Degradation and interconversion of metabolites

Analyte interconversion reactions are an issue in metabolomics, as they falsify quantitative as well as labelling results and mislead their interpretation. As an example, the measured concentration or the labelling pattern of a targeted analyte can either stem from the actual intracellular metabolite under investigation or from the chemical degradation of another component of the cell extract resulting in this metabolite or it can be the sum of both. Interconversion reactions can occur anytime between quenching of the cells and ionization in the mass spectrometer. Especially harsh conditions applying high temperature or non-aqueous and/or non-buffered conditions increase the likelihood of such chemical interconversions. We applied such conditions also in our protocol, especially during boiling ethanol cell extraction and during derivatization and injection for GC-MS. Harsh extraction conditions cannot be avoided as they were shown to be necessary to extract metabolites with high recovery from yeast[3]. Furthermore, GC-MS is still the gold standard for the separation of sugar and sugar phosphate isomers as well as other phosphorylated metabolites and demands for derivatization.

Special attention has to be paid to interconversion reactions when the unphosphorylated or decarboxylated analogue of a metabolite is targeted in quantitative or tracer analysis. As pyruvate and glycerate are of major importance in our study, as branch points for different possible pathways, we focused on these two metabolites. For tracking interconversion, we measured single standards of potential educts. The chromatograms and mass spectra of the respective degradation products are shown in Supplementary Fig. 4-6. The identity of the degradation products was confirmed by comparing them to authentic standards of the respective metabolites.

Glycerate is a metabolite involved in the serine cycle and is analysed for this study via TBDMS GC-EI-TOFMS with splitless injection. Analysis with EtOx/TMS GC-CI-TOFMS is not possible due to interference with other metabolites in the extract. As degradation of 2-PG and 3-PG was suspected due to prior results showing instability of 2-PG even in samples stored at -80°C, we investigated the stability of these metabolites with regard to glycerate formation. Our results confirm, that glycerate is, besides being an important metabolite, a degradation product of 2-phosphoglycerate (2-PG) and, to a lower extent, also of 3-phosphoglycerate (3-PG) as depicted in Supplementary Fig. 4. It has to be pointed out that although the glycerate concentration is close to LOD in the chromatograms shown in Supplementary Fig. 4, the problem is more severe, as the metabolite glycerate is low in concentration and hence measured with splitless injection (see Supplementary Data 4), while the chromatograms in Figure 9 were recorded with a 1:50 split injection. Therefore, it needs to be taken into account, that the labelling pattern of glycerate can be falsified by a contribution of the labelling patterns of 2-PG and 3-PG. By comparing the labelling degrees of the detected glycerate peak with the labelling degree of 2-PG and 3-PG, it can nevertheless be evaluated, whether the metabolite glycerate is labelled to a higher extent than 2-PG or 3-PG. If this was the case, the higher labelling degree could only stem from metabolically active pathways, in which glycerate is metabolically upstream of its phosphorylated analogues. In our case this would indicate that the natural serine cycle is active. However, this is not the case in our findings, as glycerate, 2-PG and 3-PG show similar labelling patterns. Hence the labelling pattern of the detected glycerate peak could also stem from 2-PG and 3-PG degradation only.

The problem of interconversion is even more severe if degradation products stem from two different educts, which are involved in separate pathways. This is the case for pyruvate. Pyruvate is the decarboxylation product of oxaloacetate (Supplementary Fig. 5), which is involved in the TCA cycle as well as in the oxygen tolerant reductive glycine pathway and the serine cycle pathway. Pyruvate is also the degradation product of phosphoenolpyruvate (PEP) (Supplementary Fig. 5), which is involved in glycolysis and gluconeogenesis. The pyruvate labelling pattern could therefore show contributions of up to three different labelling patterns: the actual pattern of the intracellular metabolite pyruvate, the pattern of PEP and the pattern of oxaloacetate. As it is impossible to distinguish between these contributions or quantify them, we did not evaluate the labelling pattern of pyruvate.

As can be seen in Supplementary Fig. 6, dephosphorylation of dihydroxyacetone phosphate (DHAP) to dihydroxyacetone (DHA) and glyceraldehyde phosphate (GAP) to glyceraldehyde (GA) was also observed. Therefore, also DHA and GA were not evaluated. DHAP and GAP could not be evaluated as they were either below the limit of detection or the mass spectra showed severe interferences from other constituents of the cell extracts.

As a matter of fact, such interconversion reaction could also occur in the case of other metabolites, e.g. dephosphorylation of other phosphorylated metabolites, and deamination of asparagine to aspartate[4,5]. As asparagine is always considered to be metabolically downstream of aspartate, pathway interpretations based on isotopologue distribution analysis are not hampered.

## Supplementary Note 2. Structure of the *K. phaffii* MIS genes

The cytosolic MIS gene of *K. phaffii* is split into *MIS1-3* (methylenetetrahydrofolate dehydrogenase & methenyltetrahydrofolate cyclohydrolase) and *MIS1-2* (formate-tetrahydrofolate ligase), as can be seen in Figure S3. This split results in a higher expression of the second gene *MIS1-2* (Supplementary Fig. 9). These enzymes are generally essential for native growth, as they are involved in the formation of 10-formyl-tetrahydrofolate, which is necessary for de-novo purine synthesis. Other yeasts, such as *S. cerevisiae* with *ADE3,* still have these enzymes expressed in one gene (Supplementary Fig. 11). The mitochondrial homolog *MIS1-1* in *K. phaffii* is not split (Supplementary Fig. 10). *MIS1-1* seems to be not intensely involved in de-novo purine synthesis as its knockout does not influence growth on substrates without any hypoxanthine supplementation. Nonetheless, the cytosolic version seems to be of significant importance, as when knocked out growth is not feasible unless hypoxanthine supplementation. Higher expression of formate-tetrahydrofolate ligase due to the gene separation might have given *K. phaffii* an evolutionary advantage, as more 10-formyl-tetrahydrofolate might be formed when grown on methanol via formate fixation, which would in turn lead to faster de-novo purine synthesis.

## Supplementary Note 3. Pathway efficiencies & calculated net methanol and $CO_2$ yields

These equations just consider the amount of methanol needed to fixate carbon, no maintenance. All conversions of NADH to ATP are calculated using the malate aspartate shuttle (1 NADH = 2.5 ATP), this even favors the Xu5P cycle, but also all the theoretical yields.

AOX-dissimilation: MeOH → Formaldehyde ADH-dissimilation: MeOH → Formaldehyde + 1 NADH

MeOH → Formate + 1 NADH    MeOH → Formate + 2 NADH

MeOH → $CO_2$ + 2 NADH    MeOH → $CO_2$ + 3 NADH

- **Xylulose monophosphate pathway assimilation:**

  3 Formaldehyde (C1) + 3 ATP → GAP (C3) → Pyruvate (C3) + 2ATP + NADH

  3 Formaldehyde (C1) → Pyruvate (C3) + 0.6 NADH

  With AOX net: MeOH (C1) → Pyruvate (C3) + 0.6 NADH **1 MeOH / fixed C**

  Dissimilation corrected: 2.7 MeOH (C1) + 0.3 CO2 (C1) → Pyruvate (C3) **0.9 MeOH / fixed C**

  With ADH net: MeOH (C1) → Pyruvate (C3) + 1.6 NADH **1 MeOH / fixed C**

  Dissimilation corrected: 2.47 MeOH (C1) + 0.53 $CO_2$ (C1) → Pyruvate (C3) **0.82 MeOH / fixed C**

- **Calvin-Benson-Bassham pathway assimilation:**

  3 $CO_2$ (C1) + 9 ATP + 6 NADH → GAP (C3) → Pyruvate (C3) + 2 ATP + NADH

  3 $CO_2$ (C1) + 7 ATP + 5 NADH → Pyruvate (C3)

  With AOX net: 3.9 MeOH (C1) → Pyruvate (C3) + 0.9 $CO_2$ **1.3 MeOH / fixed C**

  With ADH net: 2.6 MeOH (C1) +0.4 CO2 (C1) → Pyruvate (C3) **0.87 MeOH / fixed C**

- **Oxygen-tolerant reductive glycine pathway (enzymatic tetrahydrofolate pathway):**

  2 Formate (C1) + CO2 (C1) + 2 ATP + 3 NADH → Pyruvate (C3)

  With AOX net: 2.9 MeOH (C1) + 0.1 $CO_2$ (C1) → Pyruvate (C3) **0.97 MeOH / fixed C**

  With ADH net: 2 MeOH (C1) + 1 $CO_2$ (C1) → Pyruvate (C3) + 0.2 NADH **0.67 MeOH / fixed C**

  Dissimilation corrected: 1.93 MeOH (C1) + 1.06 $CO_2$ (C1) → Pyruvate (C3) **0.64 MeOH/ fixed C**

- **Oxygen-tolerant reductive glycine pathway (spontaneous tetrahydrofolate pathway):**

  2 Formaldehyde (C1) + CO2 (C1) + 1 NADH → Pyruvate (C3)

  With AOX net: 2.5 MeOH (C1) + 0.5 $CO_2$ (C1) → Pyruvate (C3) **0.83 MeOH / fixed C**

  With ADH net: 2 MeOH (C1) + 1 $CO_2$ (C1) → Pyruvate (C3) + 1 NADH **0.67 MeOH / fixed C**

  Dissimilation corrected: 1.67 MeOH (C1) + 1.33 $CO_2$ (C1) → Pyruvate (C3) **0.56 MeOH/ fixed C**

Net $CO_2$ fixation can only be achieved if a yield of less than 1 MeOH per fixed carbon is achieved and an $CO_2$ fixation route is present. The Calvin-Benson-Basham pathway is the least efficient pathway of the list above. Net $CO_2$ fixation to the energetic level of pyruvate can only be achieved with *ADH2*, not with AOX. The methanol yield under AOX is comparable between the xylulose monophosphate pathway and the oxygen-tolerant reductive glycine pathway via the enzymatic tetrahydrofolate pathway. Still, only the reductive glycine pathway can fixate $CO_2$ and has a yield of lower than 1 methanol to pyruvate. Under ADH the reductive glycine pathway outperforms the xylulose mono-phosphate pathway and is significantly more efficient based on these calculations with 0.67 equivalents of methanol while 0.33 equivalents of $CO_2$ are fixed into one carbon at the energetic level of pyruvate. When comparing the oxygen-tolerant reductive glycine pathway with the spontaneous tetrahydrofolate condensation with formaldehyde, no ATP is used in the enzymatic tetrahydrofolate pathway, which even leads to yields under AOX which outperforms the xylulose monophosphate pathway and reaches calculated values under ADH2 of 0.56 equivalents of methanol while 0.44 equivalents of $CO_2$ are fixed into one carbon at the energetic level of pyruvate.

## Supplementary Method 1. Strain construction-combining split marker cassettes and CRISPR/Cas9 or improved knockout-yields for the lethal *mis1-2&3* gene deletion

The mitochondrial homologous gene *mis1-1*, which encodes for formate-tetrahydrofolate ligase, methenyltetrahydrofolate cyclohydrolase and methylenetetrahydrofolate reductase, can be knocked out with the CRISPR/Cas9-based homology directed recombination. The cytosolic *mis1-2&3* version can neither be knocked out with the above mentioned CRISPR system, nor a recombination based split marker method, nor the combination of both, even if the mitochondrial homolog is still present. We screened over 600 clones with the CRISPR System, but only 3 knockouts with recombination were found. When applying the split marker, 14 knockouts out of 48 clones were detected, but all with ectopic reintegrations of the target gene. If the split marker method and the CRISPR-plasmid with Cas9 and guide RNA were transformed together and selected for both, the yield could be increased to 18 knockout clones with reintegrations out of 42 clones. We observed that the *mis1-2&3* gene can only be knocked out by supplementing YPD medium with 10 mmol $L^{-1}$ hypoxanthine, as the *mis* genes are involved in purine de-novo synthesis and a knockout of these is therefore lethal. When supplementing with hypoxanthine, 8 out of 57 clones which were transformed with the split marker system had a knockout, but only one clone without reintegration. When transforming with the the split marker system and the CRISPR-plasmid, but selecting for the split marker only, the yield was increased to 5 knockouts, with 1 out of 13 screened clones without reintegration. The best yield was achieved by combining split marker and CRISPR-plasmid and selecting for both of them (selecting with 2 resistances increased the incubation time of the transformation plate from 2 to 3 days at 30°C). 32 knockouts with 25 clones without reintegration out of 38 clones were detected. These findings show that the combination of the split marker method and the CRISPR-plasmid with Cas9 and guide RNA increases the yield of difficult or lethal knockouts. The *mis1-2&3* knockout without reintegration grew slower and could not grow when hypoxanthine was not supplemented. This knockout was also tested for capability of growth on YNB media with 18g $L^{-1}$ glycerol and 5 mmol $L^{-1}$ hypoxanthine, but growth stopped after one doubling. Additionally the doubling took 24 h, indicating that hypoxanthine is important for the mis1-2&3 knockouts but other ingredients of YPD are necessary for continuous growth.

## Supplementary Method 2. Sample preparation & GC-TOF-MS analysis of intracellular metabolites

To be able to gather all labelling data of interest by covering a high number of important metabolites, the usage of a diverse set of different GC-MS methods was necessary. Phosphorylated metabolites demand for chemical ionization due to extensive fragmentation in EI, the separation of sugars and their phosphorylated analogous is excellent using ethoximation followed by trimethylsilylation and separation on a 5% phenyl 95% methyl polysiloxane column[6], while specific fragmentation patterns of amino acids can be obtained using electron ionization and preceding tertbutyl-dimethylsilylation. Additionally, the concentration range of intracellular metabolites covers orders of magnitude and split injection as well as splitless injection were necessary for metabolites of high and low concentration, respectively, to avoid to exceed the linear range of the method. Supplementary Table 4 summarizes which samples were measured with which GC-MS methods. Supplementary

Data 3&4 list details for the GC-MS methods and Supplementary Data 2 lists in detail, which GC-MS method and which data evaluation method was finally used for data evaluation after having applied the selection criteria described in the methods section of the main manuscript.

## Supplementary references


1. Zavec, D. *et al.* Beyond alcohol oxidase: The methylotrophic yeast *Komagataella phaffii* utilizes methanol also with its native alcohol dehydrogenase Adh2. *FEMS Yeast Res.* (2021) doi:10.1093/femsyr/foab009.
2. Zamboni, N., Fendt, S. M., Rühl, M. & Sauer, U. $^{13}C$-based metabolic flux analysis. *Nat. Protoc.* **4**, 878–892 (2009).
3. Canelas, A. B. *et al.* Quantitative evaluation of intracellular metabolite extraction techniques for yeast metabolomics. *Anal. Chem.* **81**, 7379–7389 (2009).
4. Kanani, H. H. & Klapa, M. I. Data correction strategy for metabolomics analysis using gas chromatography–mass spectrometry. *Metab. Eng.* **9**, 39–51 (2007).
5. Kanani, H., Chrysanthopoulos, P. K. & Klapa, M. I. Standardizing GC–MS metabolomics. *J. Chromatogr. B* **871**, 191–201 (2008).
6. Mairinger, T. *et al.* Gas chromatography-quadrupole time-of-flight mass spectrometry-based determination of isotopologue and tandem mass isotopomer fractions of primary metabolites for $^{13}C$-metabolic flux analysis. *Anal. Chem.* **87**, 11792–11802 (2015).
