# Supplementary material for: The oxygen-tolerant reductive glycine pathway assimilates methanol, formate and CO2 in the yeast Komagataella phaffii
Source: Nat Commun. 2023 Nov 27;14:7754. doi: 10.1038/s41467-023-43610-7 (PMC10682033; doi:10.1038/s41467-023-43610-7)
Supplement: Supplementary file 2 — Description of Additional Supplementary Files [file 41467_2023_43610_MOESM2_ESM.pdf]

Description of Additional Supplementary Files

File Name: Supplementary Data 1

Description: Nucleotide sequences of synthesized genes

File Name: Supplementary Data 2

Description: List of GC-MS & data evaluation methods and samples they were applied to

File Name: Supplementary Data 3

Description: TBDMS GC-EI-TOFMS analytes, retention times, evaluated fragments (fragment structure is explained in more detail by Zamboni *et al*. [Supplementary Ref. 2]) and corresponding isotopologues & *m/z* ratios

File Name: Supplementary Data 4

Description: EtOx/TMS GC-CI-TOFMS analytes, retention times, evaluated fragments and corresponding isotopologues & *m/z* ratios
