# Supplementary material for: The oxygen-tolerant reductive glycine pathway assimilates methanol, formate and CO2 in the yeast Komagataella phaffii
Source: Nat Commun. 2023 Nov 27;14:7754. doi: 10.1038/s41467-023-43610-7 (PMC10682033; doi:10.1038/s41467-023-43610-7)
Supplement: Supplementary file 3 — Supplementray Data 1 [file 41467_2023_43610_MOESM3_ESM.docx]

## Supplementary Data 1: Nucleotide sequences of synthesized genes

Synthesized *Fhs* (of *M. extorquens*) sequence:

ATGCCATCCGATATCGAGATAGCCAGAGCAGCAACATTGAAACCCATCGCTCAGGTAGCAGAAAAGTTGGGTATTCCCGACGAGGCTTTGCACAACTATGGCAAGCATATCGCTAAAATAGATCACGACTTTATCGCAAGCTTGGAAGGAAAACCTGAGGGAAAGTTGGTATTGGTTACCGCCATATCGCCCACGCCTGCTGGTGAAGGAAAGACAACAACGACCGTTGGACTTGGAGACGCATTGAACAGGATCGGCAAAAGAGCCGTCATGTGCCTGAGAGAACCATCTCTGGGTCCTTGTTTCGGAATGAAGGGTGGAGCAGCAGGTGGTGGTAAAGCCCAGGTCGTTCCGATGGAGCAGATCAATCTCCACTTCACAGGGGATTTTCACGCCATTACCAGCGCCCACTCCCTGGCTGCCGCTTTGATAGATAACCATATTTACTGGGCTAACGAATTGAATATTGACGTACGTAGGATTCACTGGCGTAGAGTTGTAGACATGAATGATAGAGCATTAAGAGCCATTAACCAATCTCTAGGTGGAGTTGCCAATGGATTCCCGAGAGAGGATGGCTTCGATATTACAGTTGCCTCTGAGGTTATGGCCGTGTTTTGCCTGGCTAAGAACTTGGCCGACTTAGAAGAGAGGCTCGGAAGGATCGTAATTGCTGAGACAAGGGATCGTAAGCCCGTCACCCTTGCTGATGTTAAAGCCACTGGCGCAATGACAGTCCTTTTGAAAGATGCCCTACAGCCAAATCTCGTTCAGACACTAGAAGGAAACCCGGCTCTTATTCATGGCGGCCCATTTGCAAACATTGCTCATGGCTGCAACTCAGTAATTGCTACTCGAACTGGATTGCGATTAGCTGACTATACCGTGACCGAAGCTGGATTTGGCGCTGACCTAGGGGCCGAGAAATTCATCGATATTAAATGCAGACAGACCGGTTTGAAGCCCTCGGCCGTCGTGATCGTTGCTACCATTAGAGCTCTGAAGATGCACGGTGGCGTTAATAAGAAAGACCTTCAGGCTGAAAATCTGGACGCTCTAGAAAAGGGTTTCGCAAACTTAGAGCGACATGTAAATAACGTCAGGTCTTTCGGCTTACCTGTAGTAGTGGGAGTGAACCATTTCTTCCAAGACACCGACGCTGAGCACGCTCGACTGAAGGAATTGTGTCGAGACAGGCTTCAGGTTGAGGCCATTACCTGCAAACATTGGGCTGAAGGTGGCGCCGGCGCTGAGGCACTAGCTCAAGCTGTAGTTAAATTAGCCGAAGGTGAGCAGAAACCGCTTACTTTTGCATATGAAACGGAGACGAAGATAACAGACAAGATCAAAGCCATCGCTACTAAACTATACGGGGCTGCCGATATACAGATTGAATCTAAGGCTGCCACCAAGCTGGCAGGATTTGAGAAAGACGGATATGGTGGTTTACCAGTGTGTATGGCTAAAACACAATATTCCTTTTCAACCGATCCGACGCTAATGGGTGCCCCTAGCGGCCATCTTGTGTCAGTTCGAGATGTACGACTATCCGCCGGAGCAGGATTCGTTGTAGTGATCTGTGGTGAGATAATGACTATGCCAGGATTACCAAAGGTGCCCGCCGCAGACACAATACGTTTAGACGCCAATGGACAGATAGACGGATTGTTTTGA

Synthesized *FchA* (of *M. extorquens*) sequence:

ATGGCTGGTAATGAAACTATTGAAACTTTCCTTGACGGATTAGCCAGTTCCGCTCCTACTCCGGGCGGAGGAGGGGCTGCAGCTATTAGTGGTGCAATGGGAGCAGCACTGGTTTCTATGGTTTGTAACCTTACAATAGGAAAGAAGAAATATGTTGAGGTCGAGGCAGACCTAAAGCAAGTTCTTGAGAAATCCGAGGGTTTAAGACGTACCTTGACAGGAATGATAGCTGATGATGTTGAGGCTTTCGACGCAGTGATGGGCGCATACGGTTTGCCGAAGAATACAGACGAGGAAAAGGCTGCTAGGGCTGCCAAGATCCAAGAGGCACTTAAAACCGCTACAGACGTCCCATTGGCCTGCTGTCGTGTCTGCAGAGAGGTTATAGATCTAGCCGAAATTGTCGCTGAGAAGGGTAACTTAAACGTCATATCAGACGCTGGTGTCGCTGTCCTCAGTGCCTACGCCGGACTTCGTTCCGCTGCCCTTAATGTTTACGTTAACGCTAAGGGACTTGATGATCGTGCTTTCGCCGAGGAGAGACTGAAAGAATTGGAAGGGCTTCTGGCAGAGGCCGGTGCACTGAATGAAAGGATTTATGAAACCGTCAAATCTAAGGTCAACTGA

Synthesized *MtdA* (of *M. extorquens*) sequence:

ATGAGTAAGAAGTTGTTATTTCAGTTCGATACTGACGCCACTCCCTCTGTTTTCGACGTTGTCGTTGGTTATGATGGTGGAGCCGATCACATAACAGGCTACGGTAATGTTACACCCGACAACGTTGGTGCTTACGTCGACGGCACAATCTACACCAGAGGTGGTAAAGAGAAACAGTCTACAGCTATATTCGTCGGTGGTGGAGACATGGCCGCAGGCGAAAGAGTGTTCGAGGCTGTAAAGAAACGTTTCTTTGGTCCGTTTAGAGTGAGTTGCATGCTTGACTCAAATGGTAGTAATACCACGGCAGCAGCTGGTGTAGCCCTAGTAGTCAAAGCAGCTGGTGGCAGTGTTAAAGGGAAGAAGGCAGTCGTATTGGCCGGAACTGGCCCAGTTGGGATGCGTTCTGCCGCCCTGCTCGCCGGCGAGGGCGCTGAGGTTGTGTTGTGTGGCCGTAAGTTAGACAAAGCCCAAGCTGCTGCCGACTCAGTTAATAAAAGATTCAAGGTCAATGTAACTGCCGCCGAAACCGCCGATGACGCCTCGCGAGCCGAGGCCGTCAAGGGCGCTCACTTCGTGTTTACTGCCGGTGCTATCGGTCTGGAACTGTTACCACAAGCTGCTTGGCAGAACGAATCTTCAATTGAAATCGTTGCCGACTACAACGCCCAACCACCTTTGGGAATAGGTGGTATTGACGCTACTGACAAGGGCAAGGAGTACGGTGGCAAGCGAGCATTCGGAGCTCTGGGAATCGGTGGCCTCAAATTGAAGTTGCACCGTGCCTGTATCGCAAAATTATTTGAGTCATCTGAGGGTGTGTTTGATGCAGAAGAAATTTACAAGTTAGCTAAAGAGATGGCATAA
