# Supplementary material for: The oxygen-tolerant reductive glycine pathway assimilates methanol, formate and CO2 in the yeast Komagataella phaffii
Source: Nat Commun. 2023 Nov 27;14:7754. doi: 10.1038/s41467-023-43610-7 (PMC10682033; doi:10.1038/s41467-023-43610-7)
Supplement: Supplementary file 7 — Reporting Summary [file 41467_2023_43610_MOESM7_ESM.pdf]

nature portfolio

Corresponding author(s): Diethard Mattanovich

Last updated by author(s): Aug 15, 2023

# Reporting Summary

Nature Portfolio wishes to improve the reproducibility of the work that we publish. This form provides structure for consistency and transparency in reporting. For further information on Nature Portfolio policies, see our Editorial Policies and the Editorial Policy Checklist.

## Statistics

For all statistical analyses, confirm that the following items are present in the figure legend, table legend, main text, or Methods section.

| n/a | Confirmed | |
|---|---|---|
| ☐ | ☒ | The exact sample size (*n*) for each experimental group/condition, given as a discrete number and unit of measurement |
| ☐ | ☒ | A statement on whether measurements were taken from distinct samples or whether the same sample was measured repeatedly |
| ☒ | ☐ | The statistical test(s) used AND whether they are one- or two-sided<br>*Only common tests should be described solely by name; describe more complex techniques in the Methods section.* |
| ☒ | ☐ | A description of all covariates tested |
| ☒ | ☐ | A description of any assumptions or corrections, such as tests of normality and adjustment for multiple comparisons |
| ☐ | ☒ | A full description of the statistical parameters including central tendency (e.g. means) or other basic estimates (e.g. regression coefficient) AND variation (e.g. standard deviation) or associated estimates of uncertainty (e.g. confidence intervals) |
| ☒ | ☐ | For null hypothesis testing, the test statistic (e.g. *F*, *t*, *r*) with confidence intervals, effect sizes, degrees of freedom and *P* value noted<br>*Give P values as exact values whenever suitable.* |
| ☒ | ☐ | For Bayesian analysis, information on the choice of priors and Markov chain Monte Carlo settings |
| ☒ | ☐ | For hierarchical and complex designs, identification of the appropriate level for tests and full reporting of outcomes |
| ☒ | ☐ | Estimates of effect sizes (e.g. Cohen's *d*, Pearson's *r*), indicating how they were calculated |

*Our web collection on statistics for biologists contains articles on many of the points above.*

## Software and code

Policy information about availability of computer code

| | |
|---|---|
| Data collection | Agilent Technologies MassHunter Workstation GC/MS Data Aquisition Software 10.0.384.1 SR1 |
| Data analysis | Mass Spectrometry data were analyzed with Agilent Technologies MassHunter Workstation Quantitative Analysis for TOF Build 10.1.733.0 and Isotopologue Correction Toolbox Version 0.04 |

For manuscripts utilizing custom algorithms or software that are central to the research but not yet described in published literature, software must be made available to editors and reviewers. We strongly encourage code deposition in a community repository (e.g. GitHub). See the Nature Portfolio guidelines for submitting code & software for further information.

## Data

Policy information about availability of data

All manuscripts must include a data availability statement. This statement should provide the following information, where applicable:

- Accession codes, unique identifiers, or web links for publicly available datasets
- A description of any restrictions on data availability
- For clinical datasets or third party data, please ensure that the statement adheres to our policy

All data generated or analysed during this study are included in this published article (and its Supplementary information files). Raw data and processed data are available from the corresponding author on reasonable request.

As Database for Gene Annotations NCBI Blast (https://blast.ncbi.nlm.nih.gov/Blast.cgi), Pichia Genome database (http://pichiagenome-ex.boku.ac.at), Saccharomyces genome database (https://www.yeastgenome.org/) were used.

## Research involving human participants, their data, or biological material

Policy information about studies with human participants or human data. See also policy information about sex, gender (identity/presentation), and sexual orientation and race, ethnicity and racism.

| | |
|---|---|
| Reporting on sex and gender | N/A |
| Reporting on race, ethnicity, or other socially relevant groupings | N/A |
| Population characteristics | N/A |
| Recruitment | N/A |
| Ethics oversight | N/A |

Note that full information on the approval of the study protocol must also be provided in the manuscript.

# Field-specific reporting

Please select the one below that is the best fit for your research. If you are not sure, read the appropriate sections before making your selection.

☒ Life sciences ☐ Behavioural & social sciences ☐ Ecological, evolutionary & environmental sciences

For a reference copy of the document with all sections, see nature.com/documents/nr-reporting-summary-flat.pdf

# Life sciences study design

All studies must disclose on these points even when the disclosure is negative.

| | |
|---|---|
| Sample size | Sample size or clone number was n=2, if not specified differently in the figure legend or caption. no sample size calculation was performed, number of replicates relates to counting. The sample sizes were based on former studies by Mairinger et al. cited in the Manuscript. |
| Data exclusions | No data was excluded |
| Replication | All attempts at replication were successful. growth experients were replicated in different amounts which can be seen in supplementary and main text. Depending on strains in different amounts. Mass Spec experiments were not replicated due to accuracy investigated by Mairinger et al. cited in the Manuscript. |
| Randomization | Experimental groups were allocated based on their genotype and cultivation condition (e.g. carbon source); samples of labelling experiments were randomized during metabolite extraction and the GC-MS measurement sequence |
| Blinding | Blinding was not performed in this study because not all samples were measured by all GC-MS methods and samples had to be distinguishable during sample preparation after extraction. |

# Reporting for specific materials, systems and methods

We require information from authors about some types of materials, experimental systems and methods used in many studies. Here, indicate whether each material, system or method listed is relevant to your study. If you are not sure if a list item applies to your research, read the appropriate section before selecting a response.

### Materials & experimental systems

| n/a | Involved in the study | |
|---|---|---|
| ☒ | ☐ | Antibodies |
| ☐ | ☒ | Eukaryotic cell lines |
| ☒ | ☐ | Palaeontology and archaeology |
| ☒ | ☐ | Animals and other organisms |
| ☒ | ☐ | Clinical data |
| ☒ | ☐ | Dual use research of concern |
| ☒ | ☐ | Plants |

### Methods

| n/a | Involved in the study | |
|---|---|---|
| ☒ | ☐ | ChIP-seq |
| ☒ | ☐ | Flow cytometry |
| ☒ | ☐ | MRI-based neuroimaging |

## Eukaryotic cell lines

Policy information about cell lines and Sex and Gender in Research

| | |
|---|---|
| Cell line source(s) | Pichia pastoris (Komagataella phaffii) CBS7435 (NRRL Y-11430) was derived from NRRL culture collection, USDA, Peoria, IL U.S.A. |
| Authentication | Produced cell lines of this study were authenticated by colony PCRs with specific binding primers to identify the genotypic induced changes |
| Mycoplasma contamination | Only yeasts were used in this study and all cultivations were performed with animal-free components, therefore no mycoplasma testing was requested |
| Commonly misidentified lines (See ICLAC register) | No misidentified cell lines were used in this study |
